# Important Role of Overland Flows and Tile Field Pathways in Nutrient Transport

**DOI:** 10.1021/acs.est.3c03741

**Published:** 2023-10-23

**Authors:** Luwen Wan, Anthony D. Kendall, Sherry L. Martin, Quercus F. Hamlin, David W. Hyndman

**Affiliations:** † Department of Earth and Environmental Sciences, Michigan State University, East Lansing, Michigan 48824, United States

**Keywords:** nitrogen, phosphorus, nutrient loading, sources and pathways, groundwater, septic plumes, overland flow, tile drainage, nutrient modeling

## Abstract

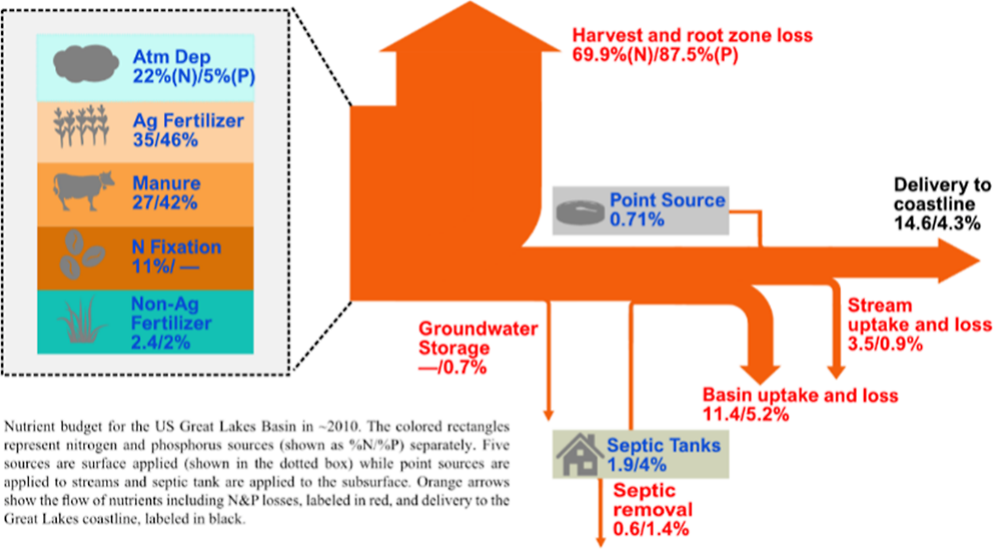

Nitrogen and phosphorus pollution is of great concern to aquatic life and human well-being. While most of these nutrients are applied to the landscape, little is known about the complex interplay among nutrient applications, transport attenuation processes, and coastal loads. Here, we enhance and apply the Spatially Explicit Nutrient Source Estimate and Flux model (SENSEflux) to simulate the total annual nitrogen and phosphorus loads from the US Great Lakes Basin to the coastline, identify nutrient delivery hotspots, and estimate the relative contributions of different sources and pathways at a high resolution (120 m). In addition to in-stream uptake, the main novelty of this model is that SENSEflux explicitly describes nutrient attenuation through four distinct pathways that are seldom described jointly in other models: runoff from tile-drained agricultural fields, overland runoff, groundwater flow, and septic plumes within groundwater. Our analysis shows that agricultural sources are dominant for both total nitrogen (TN) (58%) and total phosphorus (TP) (46%) deliveries to the Great Lakes. In addition, this study reveals that the surface pathways (sum of overland flow and tile field drainage) dominate nutrient delivery, transporting 66% of the TN and 76% of the TP loads to the US Great Lakes coastline. Importantly, this study provides the first basin-wide estimates of both nonseptic groundwater (TN: 26%; TP: 5%) and septic-plume groundwater (TN: 4%; TP: 2%) deliveries of nutrients to the lakes. This work provides valuable information for environmental managers to target efforts to reduce nutrient loads to the Great Lakes, which could be transferred to other regions worldwide that are facing similar nutrient management challenges.

## Abstract 2

Minimal research exists on spatially explicit modeling of joint surface and subsurface nutrient transport pathways. This study simulates nitrogen and phosphorus loading from sources through pathways to hotspots with a high-resolution spatially explicit nutrient transport model developed for the US Great Lakes Basin.

## 1 Introduction

Nitrogen and phosphorus loading has been linked to degraded surface water quality and the eutrophication of many coastal ecosystems worldwide. Research has focused on several ecosystems, including the Gulf of Mexico and the Chesapeake Bay in the United States,^1,2^ the Laurentian Great Lakes Basin in North America,^3,4^ as well as Taihu Lake and the Yangtze River Basin in China.^5−10^ Actions have been taken to restore and protect the water quality. For example, the United States and Canada signed the Great Lakes Water Quality Agreement in 1972 and updated it in 2012 with reduced phosphorus load targets.^3^ Although point source loads have been curtailed under the US Clean Water Act (CWA), nutrient pollution is still one of the most widespread, costly, and challenging environmental problems in the United States.^11,12^ This is partly due to the technical difficulties inherent in predicting the complex transport of pollutants from millions of “nonpoint” sources through heterogeneous hydrologic systems to receiving water bodies via diverse surface and groundwater pathways. Developing effective management and mitigation strategies requires an improved understanding of the relative contributions of different nutrient sources and their varied transport pathways.

Nutrient loading to coastal waters is derived from both point sources (primarily wastewater treatment plants) and nonpoint sources (including agricultural and nonagricultural fertilizer, manure, nitrogen fixation, and atmospheric deposition).^6,13−16^ Agricultural practices are major contributors to nutrient contamination because of the widespread use of fertilizers and livestock manure.^17,18^ Thus, managing agricultural sources typically has been the focus of efforts to reduce nitrogen and phosphorus losses to the environment.^8,9,19,20^ In contrast to the large body of research on agricultural nutrients, pollution from intensively managed urban landscapes is of great concern but is understudied.^21−23^ For example, golf courses have been cited as significant sources of nutrient loading to water bodies.^24^ Human wastewater sources may contribute more than 6 million metric tons of total anthropogenic nitrogen to the coastal ecosystems, which is roughly 45% of nitrogen delivered from agricultural areas.^25^ Septic sources are direct and concentrated inputs to the groundwater system and are substantial sources of groundwater nitrate in the United States.^26^ Septic tanks have been considered as the primary cause of nutrient leaching to groundwater because of inappropriate site conditions, poor design, inadequate maintenance, and infrequent inspections.^27^ Unfortunately, septic systems are commonly only evaluated at the time of permitting or during major building additions; less attention and resources are typically directed to septic system upkeep and maintenance, which are regulated in different ways across the United States.^28^ Modeling nutrient loading from septic tanks is challenging due to the complex and heterogeneous behavior of nutrients in the unsaturated zone and saturated groundwater systems as well as the uncertainty of the flow path of septic effluents.^28^

It is also important to know the relative importance of different pathways through which nutrients travel to receiving water bodies as transport and uptake processes differ along those pathways. Most studies have focused on pathways like surface runoff, while there has been little literature that comprehensively quantifies the relative contribution of other nutrient transport pathways.^29^ Specifically, tile drainage^30−32^ and groundwater^33−35^ are important transport pathways and major contributors to nutrient loads. For example, groundwater discharge likely accounts for ∼50% of phosphorus loaded to Lake Arendsee, Germany, thus accelerating the eutrophication of the lake and detrimental ecological impacts.^36^ Smith et al. (2015)^37^ estimated that 49% of soluble P and 48% of total P losses from fields occurred via tile discharge in research fields across the Lake Erie basin. Therefore, it is important to quantify the relative contributions of nutrients from these major pathways jointly at the surface and subsurface.

It is not feasible to measure the relative contributions of different nutrient sources and pathways at the regional watershed scale; thus, modeling approaches have been used to simulate their fate and transport. Three main categories of models are regression-based empirical, process-based flow and transport, and hybrid empirical and process-based models.^38^ Regression-based models are less complicated and easier to implement, although most ignore spatially explicit sources and lack mechanistic components, interactions between sources, and some nutrient loss processes.^39^ Examples of process-based models include Nutrient Export from WaterSheds (NEWS)^40,41^ and the widely used Soil and Water Assessment Tool (SWAT).^42^ NEWS simulates hydrological processes on land and subsequently nutrient transport through surface and subsurface waters.^43^ SWAT simulates transport using hydrologic response units, which lump all similar land uses, soils, and slopes within a subbasin. Hybrid empirical and process-based models such as the SPAtially Referenced Regression On Watershed attributes (SPARROW), a GIS-based watershed model developed by the United States Geological Survey (USGS), uses a hybrid approach to estimate nutrient sources, transport, and loadings around the world.^3,44−47^ In summary, existing models (i.e., SWAT, SPARROW) consider nutrient sources and retention on landscape and stream networks to predict nutrient and source contributions^48^ at the basin/watershed scale. For instance, SPARROW predicts nutrient loadings and sources for tributaries with an area higher than 150 km^2^ and further provides the ranking of sub-basins within these tributaries.^49^ In other words, the models do not characterize spatially explicit sources nor extensively model different nutrient attenuation pathways and thus provide nutrient loadings, sources, pathways, and hotspots at each cell level, which are the advantages of Spatially Explicit Nutrient Source Estimate and Flux (SENSEflux) modeling.

To address these limitations, we enhanced the spatially explicit hybrid SENSEflux model to estimate the fate and transport of nitrogen and phosphorus that originate from point and nonpoint sources. SENSEflux models transport nutrients across the landscape, stream network, and connected inland lakes to the Great Lakes coastline via spatially explicit pathways, including overland flow, tile drains, groundwater, and septic plumes. An earlier version of SENSEflux was developed for the Lower Peninsula of Michigan;^50^ here, additional modeling capabilities for nutrient reduction and retention processes have been incorporated. In particular, SENSEflux offers an improved simulation of nutrient delivery to streams via groundwater pathways,^51^ estimates the long-term storage of phosphorus in the subsurface, and improves the parametrization of both in-stream and lake nutrient losses. Here, we estimate total phosphorus (TP) and total nitrogen (TN) loads from the US Great Lakes Basin (US-GLB) to the coastline and compare the relative contributions of nutrient sources, with an emphasis on transport pathways. The objective of this study is to address three questions: (1) how much TN and TP are transported to the Great Lakes annually from their US drainage basins? (2) On an annual basis, where are the nutrient delivery hotspots located? (3) What are the annual contributions of sources and pathways to nutrient transport and delivery to the Great Lakes? The results can be particularly useful for stakeholders to identify hotspot areas (e.g., high nutrient flux and yields) and major sources and pathways that contribute to nutrient inputs to the Great Lakes. This knowledge can help prioritize locations and strategies for nutrient reduction and provide valuable inputs to other hydrological and ecological studies. The SENSEflux model could be applied to other regions around the world that have nutrient management issues.

## 2 Method

### 2.1 SENSEflux Model Description

**Figure 1:**
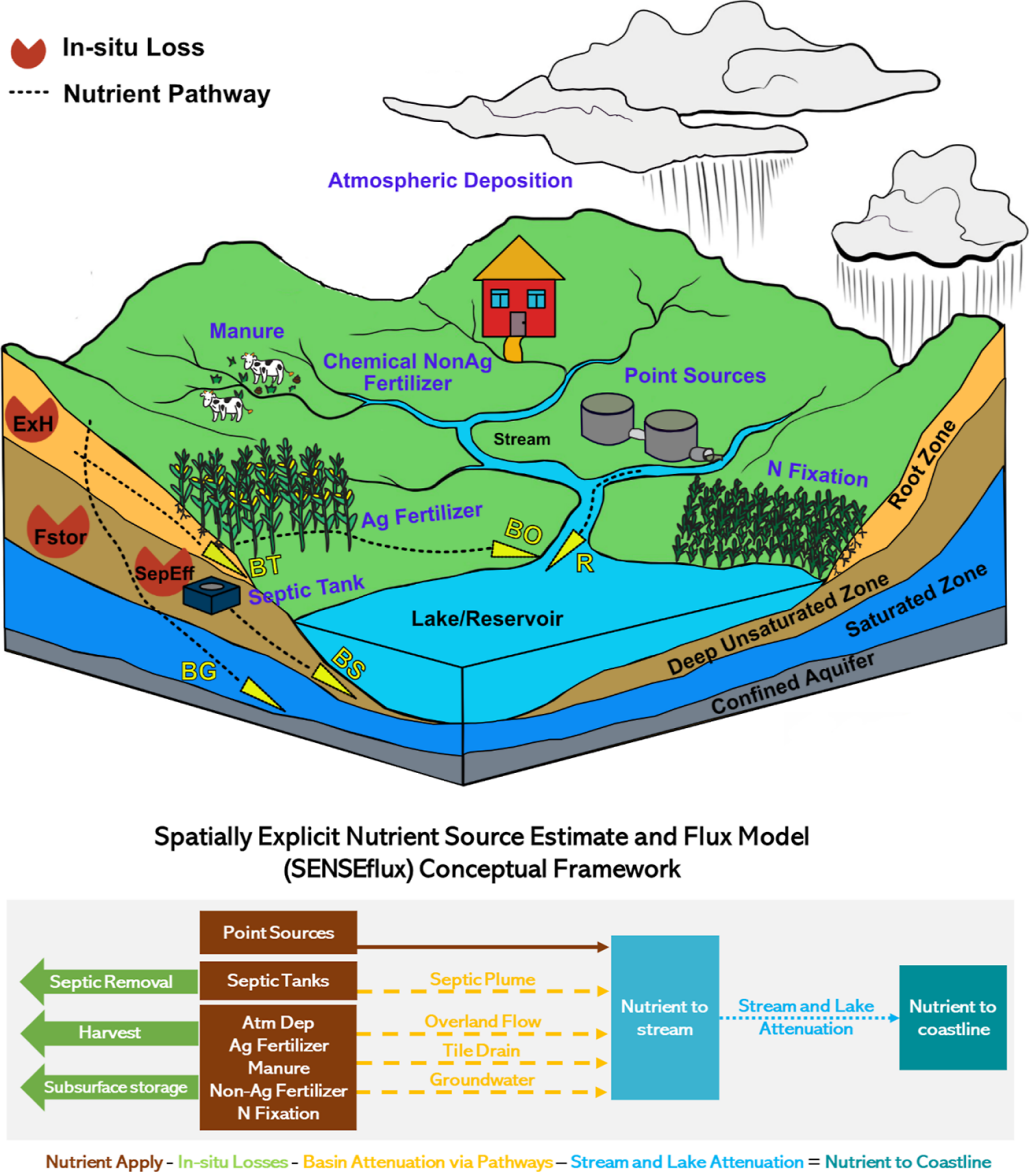
Schematic diagram (above) and conceptual framework (below) of the SENSEflux model. In the schematic diagram, seven nutrient sources are indicated in the blue text. Nutrient transport across the landscape via surface and groundwater pathways is indicated with black dashed lines and yellow triangles. Basin attenuation terms: *BO*: overland flow, *BT*: tile field, *BG*: groundwater flow, *BS*: septic plume. River attenuation term: *R*: in-stream and lake attenuation. In the conceptual diagram, brown boxes represent nutrient sources, and green arrows pointing left are septic onsite removal, crop harvest, and loss in the deep unsaturated zone. Dashed yellow arrows indicate distinct nutrient transport pathways.

SENSEflux uses a GIS and mass balance approach to simulate the nutrient fate and transport from point and nonpoint sources across the landscape through rivers to lakes and wetlands. A schematic diagram and a detailed conceptual framework of SENSEflux are shown in Figure 1. Broadly, SENSEflux includes four components: (1) nutrient applications, (2) in situ losses, (3) basin attenuation via surface and subsurface pathways, and (4) stream and lake attenuation. Loss and attenuation terms are generally spatially explicit, the product of both static landscape factors and an independent calibrated parameter for each process and nutrient. Prior to transport, three in situ loss terms remove nutrients: crop harvest and in situ loss, septic system nutrient removal efficiency, and unsaturated zone nutrient storage. We split basin transport into four distinct pathways that are not commonly represented in most hybrid and statistical nutrient transport models: overland flow, tile drainage, bulk groundwater flow, and septic plumes. Transport along each pathway includes an attenuation factor proportional to the flow distance, calibrated, and validated at sampling locations. Following basin attenuation, nutrients are then subject to stream and lake attenuation before ultimately reaching the desired end point (e.g., a sampling location or the Great Lakes coastline). SENSEflux model equations are described in the Supporting Information (Section S1).

SENSEflux supports 6 (P) and 7 (N) spatially explicit nutrient applications. There are three agricultural terms: manure, agricultural chemical fertilizers, and nitrogen fixation. Urban land use terms include chemical nonagricultural fertilizers, point sources, and septic tanks. Atmospheric deposition of both N and P occurs in all landscapes. Importantly, we do not use Net Anthropogenic Nitrogen Inputs (NANI)^52^ or Net Anthropogenic Phosphorus Inputs,^53^ though these could be computed from the SENSEflux outputs. For this study, nutrient inputs are described by the 2010 SENSEmap (Spatially Explicit Nutrient Source Estimate Map) product (described in Section 3.2).

There are three in situ loss terms applied before nutrients are transported: septic removal, harvest, and subsurface storage. The harvest (*ExH* in Figure 1) loss term includes all in-place root zone losses of nutrients (i.e., sorption, denitrification, P mineralization, etc.) and is assumed to occur in cells with either manure or chemical agricultural fertilizers applied (see “harvested areas” in Figure S1). The storage loss term (*Fstor*) includes both in-place storage and loss of nutrients below the root zone for phosphorus. Note that *Fstor* is applied only to subsurface mobile nutrients (see below). Both harvest and subsurface storage only occur for surface-applied sources (excluding septic and point sources). Finally, septic sources are subject to septic loss (*SepEff*). The spatial distributions and equations for these loss terms are detailed in the Supporting Information (Section S2).

Prior to transport, during the calculation of in situ losses, surface-applied nutrients remaining after harvest are partitioned between surface and subsurface pathways with a spatially variable partition parameter (*F*). This produces surface- and subsurface-mobile nutrient pools within each cell. The partition parameter (*F*) is assumed to vary directly with the groundwater recharge fraction (see the recharge fraction calculation in Section S1 and “groundwater recharge” in Figure S1). Nutrients applied to septic systems are subject to septic loss and then form the septic mobile pool.

Nutrients from the mobile pools are then subject to basin transport and attenuation, consisting of movement to streams from each point on the landscape, with spatially variable and source-specific attenuation occurring along the path. Surface mobile nutrients may flow to streams via either overland flow (*BO*) or agricultural tile drains (*BT*), in areas where tile drainage exists (see the “tile drained area” in Figure S1). While little overland flow occurs in most tile-drained areas,^54^ this assumption may lead to a somewhat elevated estimate of overall tile drainage flux versus overland flow. The subsurface mobile nutrients are then transported and attenuated through a groundwater flow (*BG*). Transport and attenuation of septic-mobile (remaining after septic removal) nutrients within septic plumes (*BS*) is the other important nutrient tranport pathway in the groundwater. We separated nutrient transport along this pathway because attenuation within septic plumes occurs differently than in general groundwater flow due to the distinct chemical characteristics of septic tank effluents.^28^

Nutrients remaining after basin transport are then subject to attenuation via stream and lake processes (*R*). Point sources are applied directly to streams and lakes at this step. Stream processes (which here include flow through connected wetland systems) are assumed to be split into two components: (1) water column and sediment interface losses, and (2) losses in the hyporheic zone. Water column losses include biological nutrient uptake, followed by N denitrification, particulate phosphorus transport, and sediment burial for TP. Hyporheic zone losses may include biological TN or TP uptake, denitrification (N), and sorption/mineralization (P). Lakes are represented with a linear uptake term, proportional to the length of the nutrient flow paths that intersect lacustrine-classified wetlands (i.e., lakes). Detailed descriptions for the derivation of these in-stream and lake terms are given in the Supporting Information (Section S3).

This study builds on and renames (here, SENSEflux) the model first presented by Luscz et al. (2017),^50^ incorporating new loss terms and improving multiple parametrizations. These changes were largely necessitated by the greater spatial extent of this model (see Section 3.1) and, thus, the greater range of landscape and climate characteristics present. First, we added a subsurface in situ storage term (*Fstor*) for phosphorus. We also tested this approach for nitrogen, but as parametrized, it decreased model fit and led to unrealistic results for groundwater N transport. Second, we replaced the simplistic *R* term in Luscz et al. (2017),^50^ which also relied on a basin-yield parameter that dominated the overall stream uptake, potentially skewing results. The new *R* term includes spatially variable characteristics related to denitrification or sorption, biological uptake, and lake losses. Finally, some small adjustments were made to the overall model equation. These changes are described in the Supporting Information (Sections S1–S3).

### 2.2 Model Parameter and Uncertainty Estimation

SENSEflux is calibrated separately for N and P using observed fluxes or concentrations (here, concentrations). This study uses annual-averaged fluxes (Section 3.3) and thus represents average annual losses, attenuation, and nutrient delivery. There are 10 (N) and 11 (P) scalar parameters in the SENSEflux model; these include four loss and partition terms (*SepEff*, *ExH*, *F*, and *Fstor*) as linear multipliers, with the remaining attenuation parameters (*Bo*, *Bt*, *Bs*, *Bg*, *Dnsp*, *Bio*, and *Lacus*) as multipliers within an exponent (see Equations in S1). For N, *Fstor* is set to 0. Except for *SepEff*, all parameters are then optimized via automated parameter estimation. *SepEff* is the efficiency multiplier on septic loads and is set as 0.3 for TN (0.35 for TP) based on existing studies.^50,55,56^ While SENSEflux could be used to independently calibrate multipliers for both *SepEff* and *Bs* (septic basin attenuation), here, we did not have sufficient data density to independently calibrate both.

Here, we used MATLAB’s constrained nonlinear local-minimum optimization routine *fmincon*. The objective function for this local optimization was the mean absolute difference (error) between the base-10 log (termed MAEL) of the observed and simulated nutrient concentrations. Several different objective functions were tested, including root-mean-square residuals and root-mean-square log 10 residuals. Ultimately MAEL was selected because it more equally weighted both low- and high-concentration locations, a necessity given the wide range of nutrients across the study region (Section 3.3 and Figures S2 and S5). We also tested using MAEL with loads, as opposed to concentrations, but ultimately selected concentrations because it provided higher sensitivity to attenuation parameters. The best parameter set with the lowest MAEL was used to generate maps of total deliveries and pathways and calculate nutrient fluxes, yields, sources, and pathways.

We estimated model parameter uncertainty by conducting a global search of the parameter space and then calculating the standard deviation of best local outcomes. Global optimization was conducted with the GlobalSearch solver from MATLAB’s Global Optimization Toolbox to find a global optimum for the parameters.^57^ It is based on an optimal parameter set from local optimization. The solver uses the fmincon “interior-point” algorithm to search for the global minimum using multiple starting points. Like local optimization, the objective was to minimize MAEL. Given the complex nature of the optimization with 11 parameters, global optimization can produce many “best” parameter sets with similar objective function values. Following optimization, we selected local minimum parameter sets from the global optimization and then kept the lowest 10% of the parameter sets with the lowest (best-performing) objective function values. Then, we eliminated duplicate parameter combinations, leaving a set of unique “best-performing” parameter combinations for each of our N and P optimizations. With this unique parameter set, we mapped model outcomes and then provided an estimate of uncertainty for our modeled nutrient flux, yields, pathways, and sources at the US Great Lakes Basin scale.

## 3 Study Area and Model Inputs

### 3.1 Study Area: US Coastline of the Great Lakes Basin

The Laurentian Great Lakes encompass Lakes Superior, Michigan, Huron, Erie, and Ontario, which make up Earth’s largest liquid freshwater system, with ∼21% of the world’s and ∼80% of the United States surface freshwater supply.^58^ The US Great Lakes Basin (US-GLB) includes portions of eight US states (Illinois, Indiana, Michigan, Minnesota, New York, Ohio, Pennsylvania, and Wisconsin). Average precipitation across US-GLB varies between 500 and 1600 mm yearly (Figure S2) and annual average temperatures from 3 to 10 °C in the 2010s.^59^ Forty million residents of the United States and Canada depend on this lake system for clean drinking water.^60^

Great Lakes’ ecosystems are being threatened by climate change, invasive species, and degraded water quality because of pollutants from residential, agricultural, and industrial activities.^61−63^ Urbanization, agricultural intensification, and failing septic systems are causing contamination across the GLB. Several major cities in the southern basin (e.g., Chicago, Detroit, and Cleveland) have significant impermeable surface areas that route precipitation directly to aquatic systems via overland flow. Most agricultural areas are in the southern portion of the basin (Figure S2) and produce substantial nutrient loads to the lakes.^3^ Because of humid continental climates and broad areas with very permeable soils and high aquifer recharge, 43% of the US-GLB coast is vulnerable to groundwater-borne nutrients.^64,65^ Harmful algal blooms in the GLB have been a critical issue for millions who live in the region, resulting in negative effects on industries (e.g., fishery, tourism, aquaculture), ecology (e.g., fish kills), and public health (e.g., drinking water contamination, toxicity to pets and livestock).^66−68^

### 3.2 Nutrient Source Inputs: SENSEmap

To drive SENSEflux, we used the SENSEmap product that describes each of 7 distinct sources TN or 6 sources TP at 30 m resolution, ca. 2010. These sources include point sources, along with the nonpoint sources of chemical agricultural fertilizers, chemical nonagricultural fertilizers, manure, septic tanks, atmospheric deposition, and N fixation. SENSEmap estimates sources using GIS and statistical methods constrained by broadly available data from remote sensing, government databases, and literature.^16,69^ For this study, all seven sources were aggregated to 120 m resolution, summing from the 30 m SENSEmap values (Figures S3 and S4).

### 3.3 Calibration Data: In-Stream Nutrient Load

TN and TP loads at sampling sites (TN: 116, TP: 119) within the US-GLB, which Robertson and Saad (2011)^47^ used to calibrate the SPARROW model, were extracted to calibrate and validate the SENSEflux model (Figure S5). Here, concentrations were used for calibration and validation, and measured nutrient loads with the Fluxmaster program were used to compare with the simulated ones. Concentration data were split randomly into two sets: 70% for model calibration and 30% for validation. Robertson and Saad (2011)^47^ estimated these loads using the Fluxmaster program, which may overestimate nitrogen loads and underestimate TP due to uncertainties such as the lack of continuous measurements of concentration and streamflow.^3,70,71^ The sampling locations are not evenly distributed across the US-GLB, and they are biased toward sites where there are existing nutrient delivery concerns, which likely add some uncertainties to the model results. Nevertheless, they are the most complete data set of annual loads available for the region.

### 3.4 Basin Characteristics: Groundwater Recharge, Overland Flow Length, Harvested Areas, and Tile Drained Areas

As discussed in Section 2.1 and detailed in the Supporting Information (S1–S3), spatially variable factors affect the fate and transport of TN and TP during both landscape (basin) and in-stream transport. Groundwater recharge (Figure S1), or the amount of water percolating from the surface to the water table, is used to characterize the subsurface partition (*F*) and fraction of groundwater-pathway nutrients stored in soil and the deep unsaturated zone (*Fstor*). Overland flow length was calculated using ArcGIS Hydrology Toolbox based on the Digital Elevation Model (DEM) from the National Elevation Dataset (NED), which is used as part of the reduction factor for basin pathways. Harvested areas for TN and TP are determined by where manure or chemical agricultural fertilizers are applied. A novel tile drainage layer was calculated to evaluate whether nutrients were likely transported via overland flow or tile fields. See details for groundwater recharge and tile drainage area calculation in the Supporting Information (Section S4).

### 3.5 In-Stream/Lake Characteristics: Catchments, In-Stream Travel Time, Streambed Exchange Rate, and Lake Travel Distance

The hydrologic networks move water through catchments and along rivers, with their associated drainage basin providing a critical component to hydrologic analysis and modeling.^72^ The (∼30 m) resolution 1 arc-second DEM from the USGS NED was used to calculate flow direction and flow accumulation to generate stream networks.^73^ Watersheds were delineated by using these sampling sites as pour points in the ArcGIS 10.6 Hydrology Toolbox. TN and TP watersheds are shown in Figure S5 with corresponding loads from Robertson and Saad, 2011^47^ for each watershed.

Like the overland flow length calculation in the previous section, in-stream travel time was calculated by using the ArcGIS Hydrology Toolbox with the NED DEM and the flowlength function. For this instance, a cost raster was supplied, calculated as the time/unit distance in each cell (i.e., 1/velocity). For overland flow portions of the flow path, the cost function was set to 0, while in-stream velocities were computed from USGS gauge site data (see Supporting Information, Section S3). In-stream travel time is used to calculate the biological uptake portion of stream attenuation (Equation 5 in S1).

The N denitrification/P sorption portion of the stream attenuation functions was assumed to be driven by the rate of exchange between streamflow and the stream bed and the hyporheic zone beneath. We calculated this rate of exchange as the ratio of streambed flux and in-streamflow, as described in the Supporting Information (Section S3). Ultimately, this exchange rate (Figure S6e) is the product and ratio of multiple factors, including hydraulic conductivity (Figure S2d), slope (Figure S6a), basin yield during baseflow (Figure S6b), hydraulic radius (Figure S6c), and velocity (Figure S6d). The exchange rate is then used to calculate denitrification/sorption (Equation 6 in S1).

Travel distance in lakes was computed by providing a 0/1 cost raster to the flowlength function in ArcGIS, with values of 1 indicating lakes and 0 otherwise. This layer is used to compute lake retention (Equation 7 in S1).

## 4 Results and Discussion

**Figure 2:**
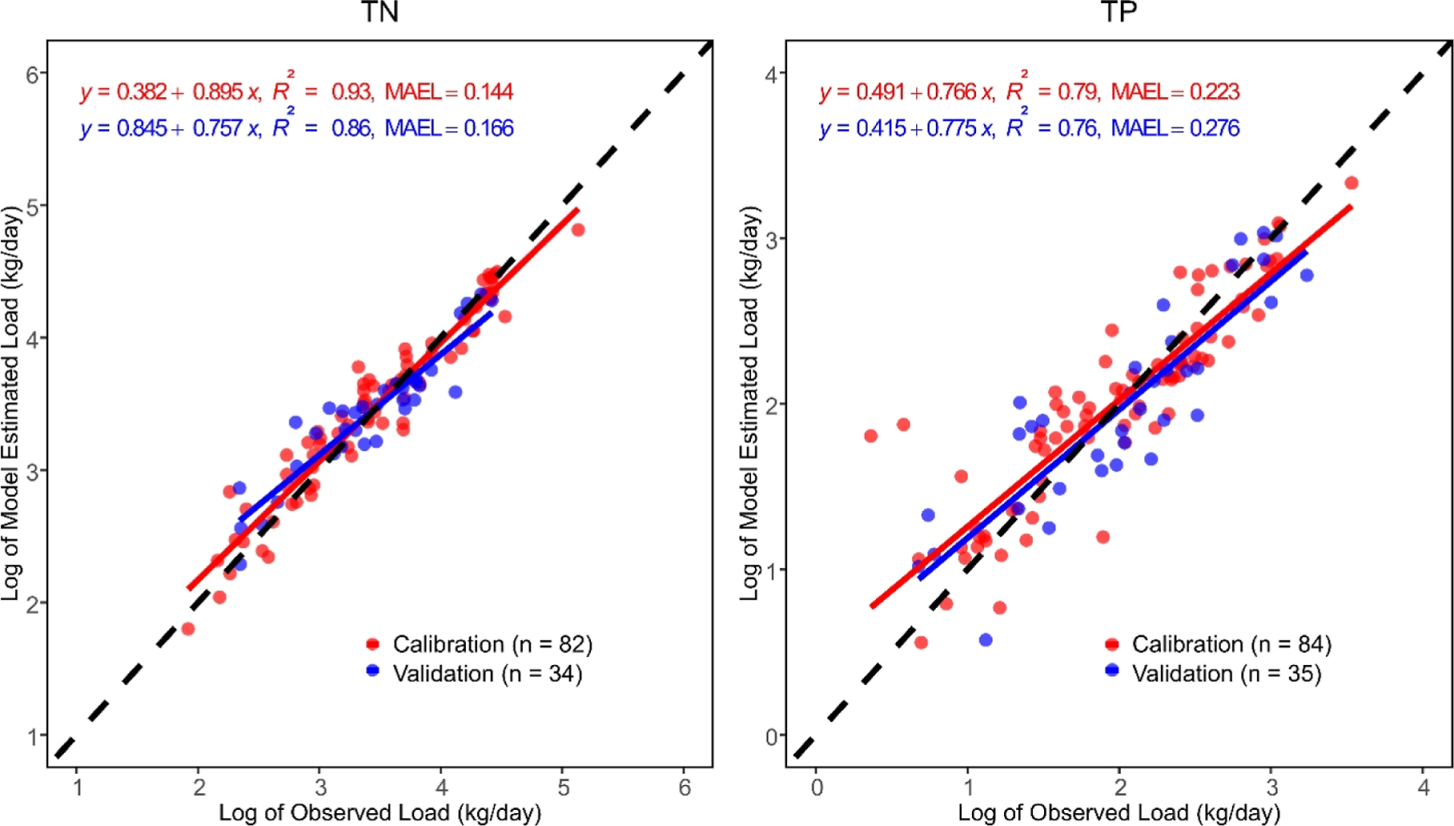
Plot of log_10_ simulated and observed daily loads for model calibration and validation data sets. The dashed black line is a 1:1 line. Solid blue and red lines indicate the regression fits. *n* refers to the number of observation points in each of the calibration and validation data sets.

The best-performing parameter set from local optimization is utilized to assess the model performance. SENSEflux TN and TP annual models performed well (calibration/validation *R*^2^ values of 0.93/0.86 and 0.79/0.76, respectively, Figure 2), providing estimates of TN and TP loads to the US-GLB using optimized parameters (Table S1). These parameters indicate higher rates of attenuation in surface vs subsurface pathways and that transport through tile drainage produces the lowest attenuation rates of all pathways (see the extended discussion in Section S5).

The TN model had a slightly better fit versus TP, with a difference of 14% for calibration and 10% for validation data sets, respectively. The best-fit line has slopes >0.75 (TN and TP calibration/validation slopes are 0.90/0.76 and 0.77/0.78, respectively), indicating a slight bias toward high predictions at low loads and low predictions at higher loads. This is especially seen in the TP model, which is likely due to an imbalanced distribution of the phosphorus data set as we have fewer high-end sites than lower values for model calibration. Overall, the model-predicted loads were close to observed values: the MAEL daily loads for TN calibration and validation are 0.14 and 0.17 log_10_ (kg/day), respectively, and 0.22 and 0.28 for TP. Analyses of residuals indicate no significant bias for TN but a slight (statistically nonsignificant) underestimate of TP deliveries for the validation data set (Figure S7). The residuals for TN and TP are not significantly different from zero with *P* values ranging from 0.4 to 0.65 based on a one-sample *t*-test (Figure S7). The spatial residuals (Figure S8) are reasonable with 83 and 77% of watersheds having residuals (log_10_) between −0.2 and 0.2 and are significantly clustered spatially with the Moran’s index of 0.43 and 0.41 for TN and TP, respectively.

Following global optimization and identification of best-performing models, the unique best-performing parameter combinations (100 for TN and 78 for TP) from 14976 TN (16841 TP) global optimization runs were selected. The minimum and maximum values from the best-performing set are reported in Table S1. Figure S9 shows the parameter uncertainties: there are larger uncertainties in TN models for *f* and *ExH* among the linear parameters. The groundwater storage parameter (*fstor*) for TP ranged from 0.49 to 0.65. For exponential parameters, *bo*, *bt*, *bg*, *bs*, *dnsp*, and *bio* have higher uncertainties in the TN model, whereas *lacus* is similarly robust in both models.

### 4.1 Spatially Varied Nutrient Delivery and Loads to Lakes

**Figure 3:**
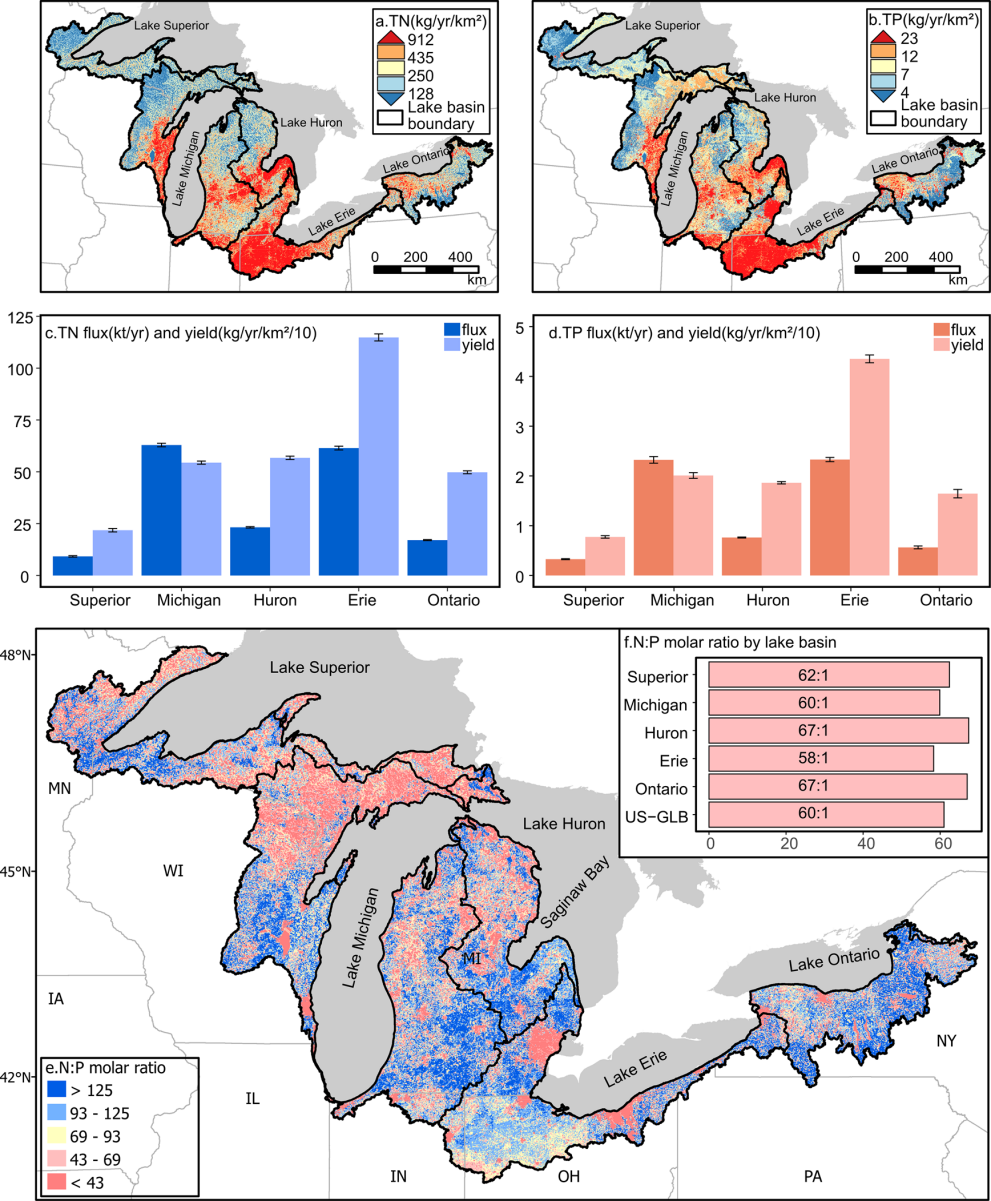
Predicted nutrient delivery as yield (kg/km^2^/yr) to the Great Lakes shoreline and summarized nutrient loads by lake basin (black outlines) within the US-GLB. TN (a) and TP (b) yields are direct outputs from SENSEflux that are resampled to 720 m resolution for display purposes and classified in quantiles within which each color represents ∼20% of the US-GLB area. Bars in [(c)—TN] and [(d)—TP] represent the total basin-accumulated fluxes and yields (area normalized) for each nutrient from the best performing parameter set within the local optimization, and error bars represent standard deviation from the unique best-performing global optimization runs. Map of N/P yield molar ratio at 720 m resolution across US-GLB, rounded to the nearest 1 (e); bar chart of mean N/P load molar ratio by lake basin within US-GLB (f).

Simulated export of TN and TP varied substantially across the US-GLB, with the majority of the area (∼60%) between 128 and 912 kg/yr/km^2^ for nitrogen and 4–23 kg/yr/km^2^ for phosphorus, encompassing the ∼20th to 80th percentile of nutrient deliveries (Figure 3a,b). Over the entire US-GLB, mean TN and TP loads are 599 and 21.7 kg/yr/km^2^, respectively. Broadly, spatial patterns are similar for both TN and TP (Pearson correlation coefficient, *r* = 0.73). For instance, both TN and TP are high in the southern Lake Michigan, Saginaw Bay, Western Lake Erie, and Lake Ontario basins (Figure 3a,b).

We also calculated the N/P ratio (ratio of total delivered TN to TP load) across US-GLB and each lake basin (Figure 3e,f), which can help us understand the drivers of lake trophic status. Overall, deliveries to the US-GLB are relatively N-enriched, with >95% (5th percentile of the ratio = 17.9, median of 80.7) of cells delivering nutrients above the classical Redfield ratio, defined as an N/P molar ratio of ∼16:1. Individual Lake basin averages of N/P delivery ratio are between ∼58 and 67 (Figure 3f). For comparison, basin averages the N/P ratios of SENSEmap nutrient inputs to the landscape from Hamlin et al. (2020)^16^ vary between ∼17 and 21, except for Lake Superior at 51.6. It is notable that in our current study, the delivery N/P ratio for Lake Superior is 62.4, indicating that the watersheds draining to this northernmost lake are relatively efficient at routing P to the coastline. In general, watershed deliveries to the lakes indicate that P should be the limiting nutrient, as is largely the case for the nonmarine waters in this region.

Lake Michigan receives the highest TN loads followed by Lake Erie, with 62.9 and 61.5 kt/yr nitrogen delivered from US lands to the water, respectively (Figure 3c and Table S2). Lake Erie has the highest TP loads followed by Lake Michigan, with 2.4 and 2.3 kt/yr phosphorus delivery (Figure 3d). These two lake basins have relatively larger uncertainties of nutrient loads, with standard deviations from the best-performing parameter set within the 10% of global optimization, which are 864 and 910 t/yr of TN (66 and 42 t/yr for TP) in Lake Michigan and Lake Erie, respectively (Figure 3c,d). Lake Huron, Ontario, and Superior have much lower deliveries, all at or below 24 kt/yr nitrogen and 0.8 kt/yr phosphorus. This is consistent with the larger US drainage basins of Lakes Michigan and Erie among the Great Lakes.

We also calculated nutrient yields, defined as nutrient fluxes divided by drainage basin area (Figure 3c,d). Not surprisingly, Lake Erie has the highest yields for both TN and TP, with nitrogen yields of 1148 kg/yr/km^2^ and phosphorus yields of 43.5 kg/yr/km^2^. Lakes Huron and Michigan have similar nitrogen yields (567 and 544 kg/yr/km^2^, respectively) and phosphorus yields (18.6 and 20.1 kg/yr/km^2^, respectively). Lake Superior has the lowest nitrogen (218.3 kg/yr/km^2^) and phosphorus yields (7.7 kg/yr/km^2^). Standard deviations of nitrogen yields from the best-performing parameter set within the 10% of global optimization are highest in the Lake Erie basin (17 kg/km^2^/yr for TN) and phosphorus yields in the Lake Ontario basin (0.84 kg/km^2^/yr) followed by Lake Erie (0.78 kg/km^2^/yr), shown in Figure 3c,d.

To evaluate the nutrient loading from SENSEflux, we compared simulated TN and TP loads with the GLB SPARROW model,^47^ which is the most comparable model with the closest time frame and is widely used as a watershed nutrient load predictor. Overall, the total modeled delivery of TN and TP from US-GLB to the lakes using SENSEflux is 0.37 and 0.45 times lower than simulated loads from the SPARROW model for TN and TP, respectively (Figure S10). More details about the reasons for these differences can be seen in the Supporting Information (Section S6).

Maccoux et al. (2016)^74^ calculated an average TP load from Lake Erie watersheds equal to 5.7 kt/yr for 2008–2012 (excluding direct point and municipal sources). Thus, the estimate presented here is roughly 42% of that from Maccoux et al. This difference is dominated by an underestimate of loads to Lake Erie from just one tributary, the Maumee River, which suggests that some important pathway or source mechanism may be missing or underestimated within SENSEflux. Future work will continue to refine nutrient input mechanisms and specifically examine varying seasonal loads—given the established importance of winter and spring deliveries in that basin.^75^

### 4.2 Nutrient Delivery Efficiency and Hotspots

**Figure 4:**
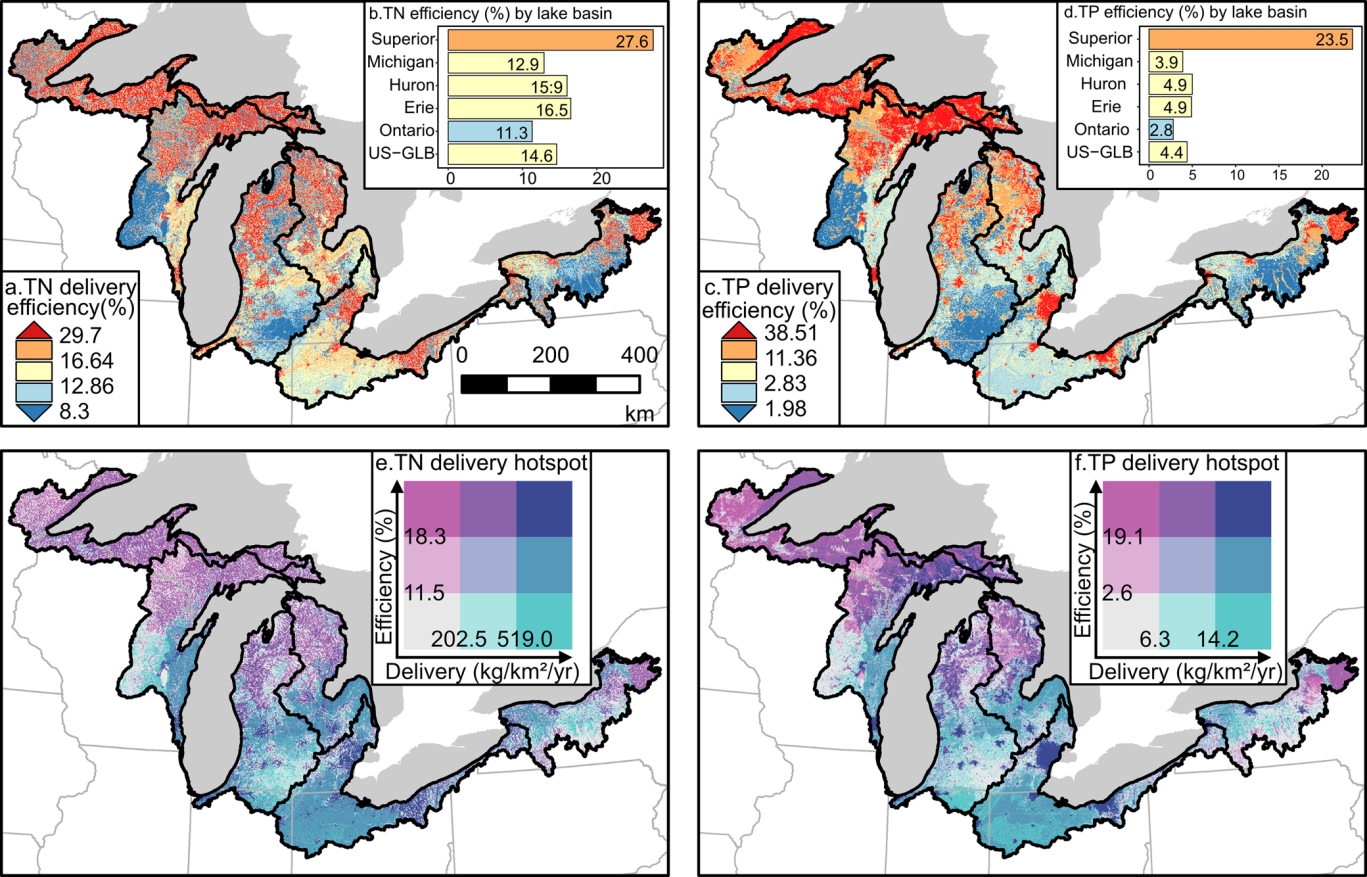
Nutrient delivery efficiency (defined as the ratio between nutrient deliveries to inputs) for TN (a) and TP (c) across the US-GLB and summaries by lake basin (b, d). Nutrient hotspots [determined as high nutrient delivery (mass of nutrients that reach the lake, *x*-axis) and delivery efficiency (proportion of input nutrients that are delivered, *y*-axis)] are shown in bivariate choropleth maps for TN (e) and TP (f).

SENSEflux provides a fully spatially explicit estimate of nutrient deliveries, allowing a novel view of the landscape: nutrient delivery efficiency, defined as the ratio of deliveries to inputs (Figure 4a,c). Median cell-by-cell TN delivery efficiency was 14.6%, while that of TP was just 3.7%. In general, northern portions of the GLB have higher delivery efficiency as do urban areas. Basin-averaged delivery efficiencies range between 11–16.5% for N and 2.8–4.9% for P in all lakes except for Superior (Figure 4b,d). There, the basin is remarkably efficient at delivering nutrients, sending almost 28% of input N and 24% of input P to the coastline. Cumulatively over the entire region, the TN delivery efficiency was 14.6% (the same as the median cell-by-cell delivery value), while the TP was 4.3% (see Graphical Abstract). Other researchers have found that 23–27% of NANI are transported to rivers and streams,^76,77^ and discrepancy in nutrient delivery efficiency (ratio between deliveries to inputs) between them and this study is likely attributed to various nutrient sources, transport mechanisms, and definitions of delivery ratio. For instance, NANI includes nitrogen sources coming from synthetic fertilizer, agricultural nitrogen fixation, atmospheric deposition, and net movement of human and animal feeds, while SENSEflux uses seven spatially explicit nitrogen sources (Section 3.2). We calculate delivery ratio using nutrient delivery to the coastline, while NANI focuses on in-stream concentrations and flux.

Deliveries are the result of both inputs and cumulative storage and attenuation processes along transport pathways. Figures S11 and S12 in the Supporting Information are maps of the loss and attenuation for N and P, respectively. Harvest is the dominant loss process across most of the domains, providing the broad N–S gradient seen in delivery efficiencies (Figure 4a, c). Stream and lake attenuation variability (Figures S11c and S12d) occurs at moderate scales, driven in large part by the travel time from the coastline. Basin transport attenuation (Figures S11b and S12c) varies at the shortest scales, responding to distance from streams, tile vs overland transport, and the presence of septic systems.

Combining nutrient deliveries (Figure 3a,b) with delivery efficiencies (Figure 4a,c) produces a novel view of landscape nutrient transport function: delivery hotspots, quantified by terciles and presented in a bivariate colormap (Figure 4e,f). Areas of the landscape with both high loading and delivery efficiency (highest 33%) are the most intense sources of loads to the coastline (shown in blue on the hotspot maps). These are predominantly urban areas, particularly for TP. Next, areas with high delivery (highest 33%) but low efficiency (lowest 33%, teal on the hotspot maps) are agricultural areas generally more distant from the coastline. Areas with low delivery (lowest 33%) but high efficiency (highest 33%) are highlighted in magenta and concentrated in the northern areas of the region. Although they do not generate substantial total deliveries, their high delivery efficiency (ratio of deliveries to inputs) makes them of conservation interest. In general, areas with high delivery efficiency would be ideal targets for conservation efforts because reducing nutrient inputs in these areas will result in a higher reduction in nutrient delivery.

### 4.3 Leading Sources of N and P Fluxes to the Great Lakes

**Figure 5:**
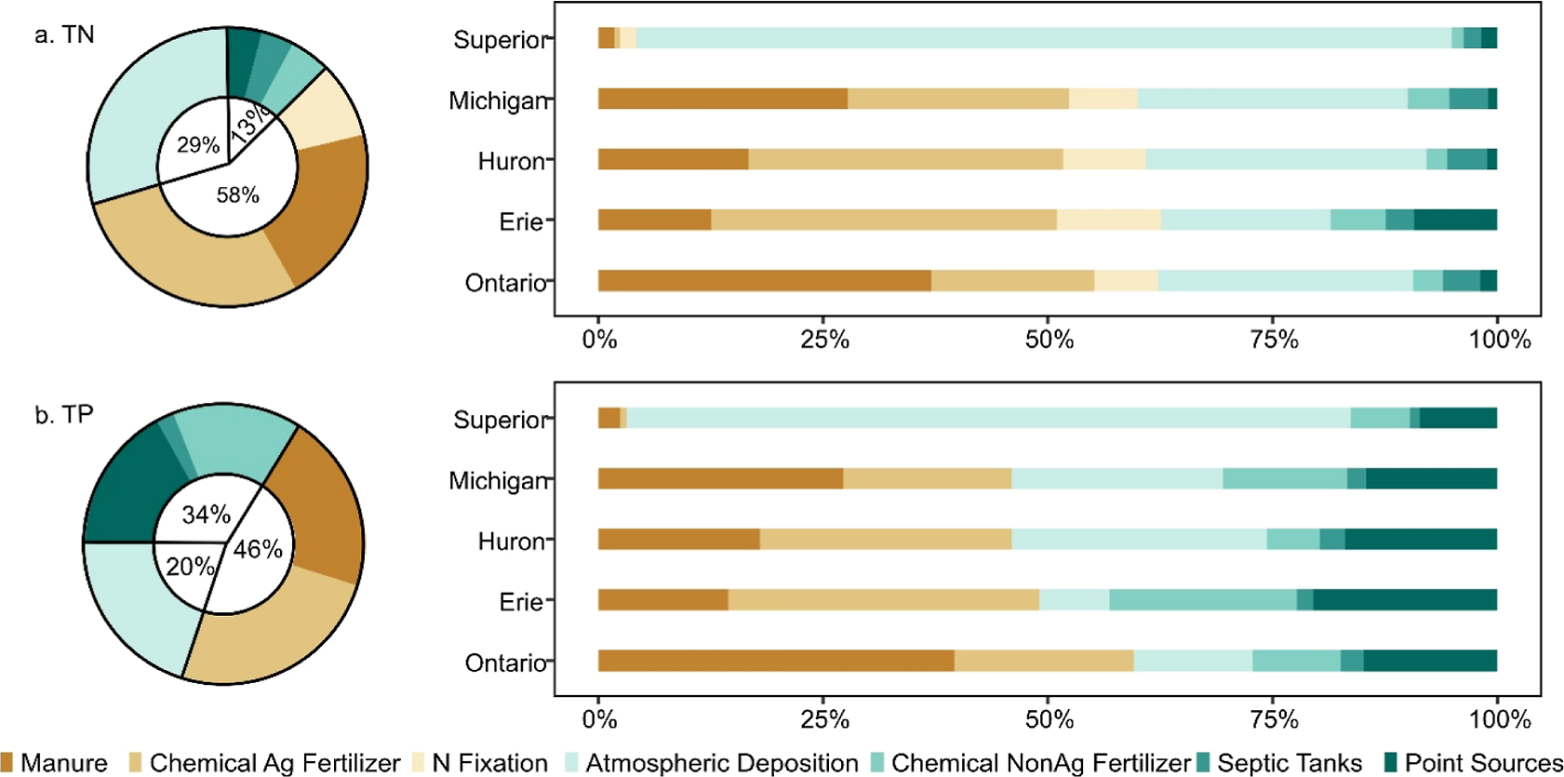
Estimated percent of nutrients delivered to lakes by source. Donut figures represent the US-GLB while rotated stacked bar plots show each lake basin within the US-GLB. The black donut circle was divided into agricultural sources (brownish), urban sources (greenish), and atmospheric deposition sources (light green) with corresponding percentages.

Agricultural sources, including manure, chemical agricultural fertilizer, and nitrogen fixation, dominate nitrogen fluxes, totaling ∼58% and up to 66% (see ranges and standard deviations of source contributions at US-GLB in Table S3 and Figure S13) of all fluxes from the US-GLB (Figure 5a). Agriculture was the largest nitrogen source of each lake, except for Lake Superior where atmospheric deposition dominated (91%, Figure 5a). The breakdown among agricultural sources is variable across lake basins. Manure was more dominant than agricultural fertilizer sources in Lakes Michigan and Ontario while agricultural fertilizer was more important in Lakes Erie and Huron. The dominant contribution of agricultural sources to nitrogen transport and delivery is consistent with the findings of Robertson and Saad (2011).^47^

Phosphorus fluxes were also dominated by agricultural sources (manure and chemical agricultural fertilizer) in the US-GLB (∼46%, Figure 5b). On the lake basin scale, phosphorus fluxes to Lakes Michigan and Ontario are driven by agricultural sources because of large manure inputs, while Lakes Erie and Huron had higher inputs from chemical agricultural fertilizer. Specifically, Lakes Michigan and Ontario had 46 and 60% phosphorus loads from agricultural sources, with manure accounting for 27 and 40% of inputs, and 19 and 20% from chemical agricultural fertilizer, respectively. For Lakes Erie and Huron, agricultural sources appear to contribute 49 and 46%, where agricultural chemical fertilizer was the dominant source (35 and 30%).

Urban sources, including chemical nonagricultural fertilizer, septic tanks, and point sources, account for only 12.5% of N contributions but 34% of total P in the US-GLB (Figure 5a,b). The contributions of these three sources for TN delivery are similar, with 4.1% of the point source, 3.8% of septic tanks, and 4.6% of chemical nonagricultural fertilizer. However, the point source accounts for 16.8% of the TP delivery. The relatively lower point source contribution for TN demonstrates the effectiveness of the CWA and the National Pollutant Discharge Elimination System permitting system. Point sources were found to be significant and contributing ∼14–44% of phosphorus and 13–34% of nitrogen delivery.^49^ The other major urban source for TP transport is chemical nonagricultural fertilizer (14.7% basin wide), which is largely applied to golf courses and lawns. This is supported by Baris et al., 2010,^78^ who found that 86% of surface water samples had phosphorus concentrations above the criteria set by the US Environmental Protection Agency, after comparing nitrate and TP concentrations from golf courses across the United States. It is critical to ensure the effective use of fertilizers in urban systems and use best management practices to reduce nutrient transport,^24^ especially for TP.

Atmospheric deposition is an important contributor of N and P, consisting of 29% TN and 20% TP sources in the US-GLB (donuts in Figure 5a,b). This is largely because of the dominant role (91% of TN, 81% of TP sources) that atmospheric deposition plays in Lake Superior, which has a severe stoichiometric imbalance with high N and low P concentrations.^79^ Unfortunately, atmospheric deposition can be difficult to manage, and research has found that current policies and technologies may not be sufficient to reduce deposition under critical loads.^80,81^

In summary, although management actions have focused on agricultural sources for decades, they still dominate TN and TP transport and delivery across most of the US-GLB. While approximately 71 and 88% of TN and TP sources applied to the landscape are from agriculture,^16^ only 58 and 46% of TN and TP are delivered to US-GLB. This means that even though a higher percentage of agricultural phosphorus source is applied to landscapes, less percentage of phosphorus is delivered to aquatic ecosystems in these lower delivery efficiency areas. These disproportional differences between the sources and fates show the importance of nutrient transport and the natural differences between TN and TP attenuation.

### 4.4 Nutrient Delivery Pathways: the Dominant and the Underappreciated

**Figure 6:**
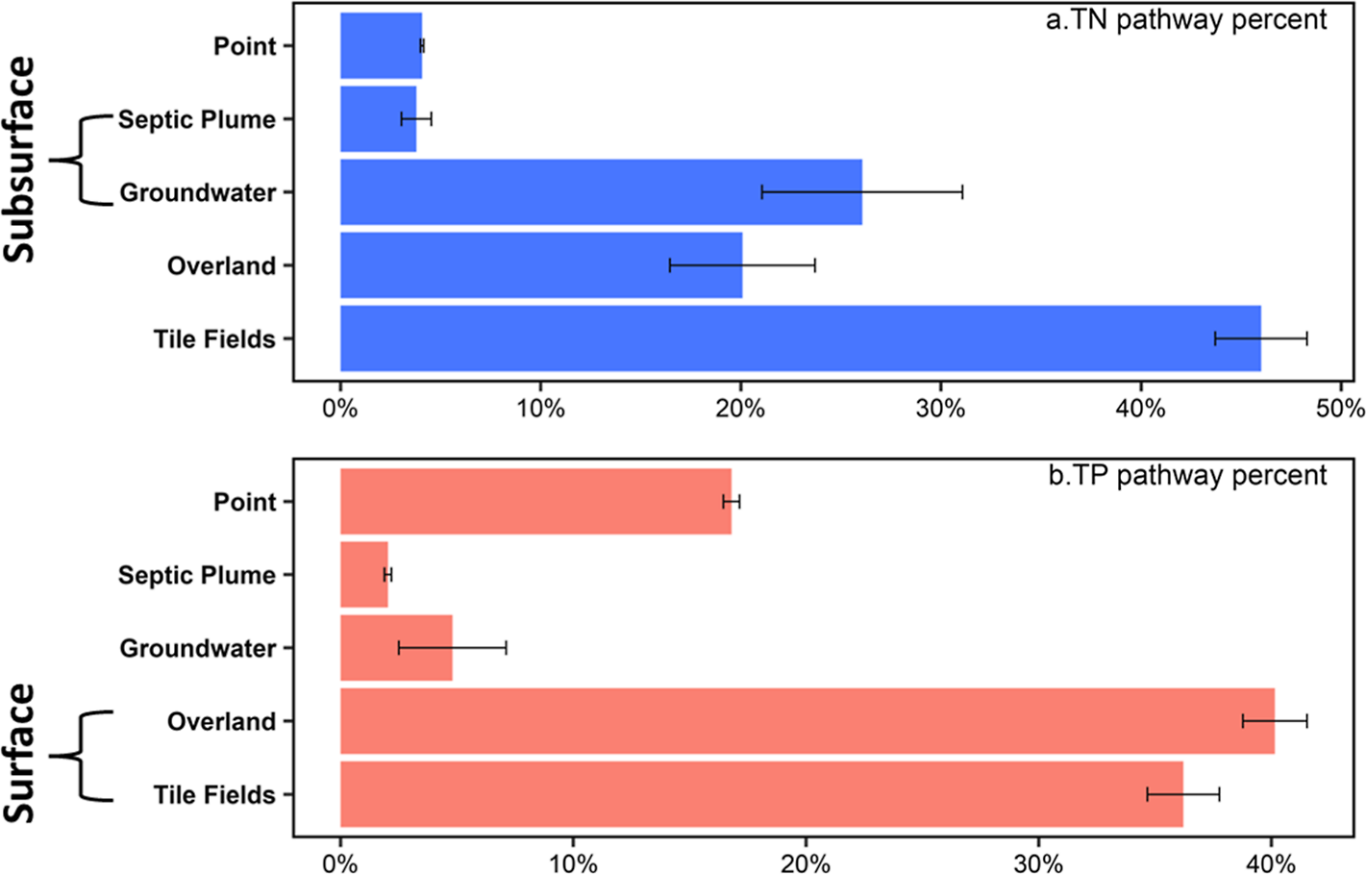
Estimated percentages of total nutrients delivered to the Great Lakes through each pathway (surface: overland and tile fields; subsurface: groundwater and septic plume). Colored bars indicate the best-performing local optimization outcome, and error bars represent parameter uncertainty given by the standard deviation from the best-performing global optimization runs.

We summed nutrient deliveries by transport pathways across the US-GLB, shown ranked from highest to lowest proportion [surface (tile fields + overland) > subsurface (groundwater + septic plume) > point] for TN and TP within the US-GLB (Figure 6). The ranges of pathway contributions at US-GLB are shown in Table S4. Our results agree with prior work that indicates surface pathways dominate transport,^82−84^ contributing the largest proportion of TN and TP (66 and 76%) delivery to the lakes. Others have shown that overland flow was the primary export pathway for both P and N, but tile drainage cannot be overlooked^37,85^ and contributes almost 50% of some TP loads.^37^ We found that overland flow was the primary pathway (40%) for delivery of TP to the US Great Lakes. TN was dominated by tile fields (46%) and 36% of TP was transported by tile fields, showing that tile drainage delivers large quantities of nutrients, especially nitrogen, to the Lakes and is thus critical to manage.

Subsurface pathways (groundwater flow and septic plumes) transport a significant proportion of nutrients to the lakes, with about 30% TN and 6.8% TP (Figure 6). The groundwater flow pathway dominates the subsurface transport of nitrogen (26% of total transport), likely due to nitrogen’s high mobility. We found that septic plumes contributed 3.8% of TN (2% of TP) to lake loading. Other studies have indicated that septic systems are important nutrient sources,^86,87^ yet they are rarely accounted for and commonly overlooked. The pathway proportions from the point sources are 4.1% for TN and 16.8% for TP, demonstrating that more efforts could reduce phosphorus loads in the Great Lakes from these sources. Note that our maps do not include direct discharges of point sources to the Great Lakes coastline.

### 4.5 Heterogeneous Pathways and Uncertainties

**Figure 7:**
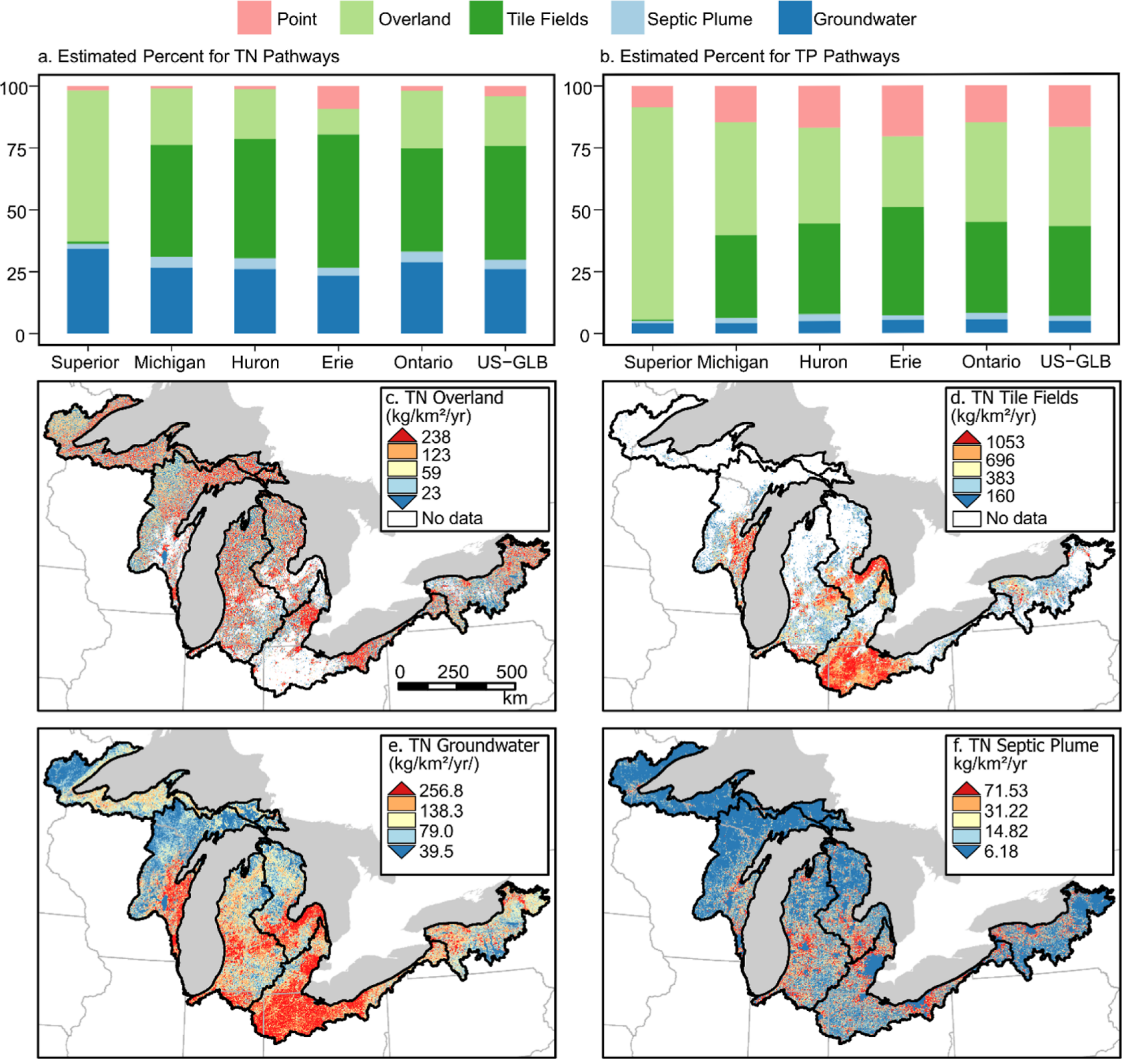
Estimated total yield of TN was delivered to lakes by four key pathways. (a,b) TN and TP pathways by lake basin; (c–f) TN overland, tile fields, groundwater, and septic plume, respectively. Maps are resampled from 120 m SENSEflux outputs to 720 m resolution here for display purposes and classified in quantiles, with each color representing 20% of the data set; the white area in (c,d) within the basin boundary represents areas with no data as we assumed that overland and tile fields are alternative pathways. Corresponding maps for TP are included in Figure S14.

Surface pathways dominated nutrient contributions within each lake basin (Figure 7a,b). In the Lake Superior basin, overland flow dominates nutrient transport, with 61% TN and 86% TP. In the Lake Michigan basin, tile drainage transported 45% TN (23% by overland flow) and 33% TP (46% by overland flow). Tile fields delivered more nutrients than overland flow in the Lakes Erie and Huron basins. This supports earlier work that found tile drainage to be the primary pathway for nutrient delivery to streams in the western Lake Erie basin.^37,88^ In the Lake Ontario basin, tile fields transported 42% nitrogen (23% for overland) and 37% phosphorus (40% for overland).

Subsurface pathway (septic plume and groundwater) contributions varied across the five lake basins (Figure 7a,b). The bulk groundwater flow pathway (excluding septic plumes) contributed substantially across the lake basins, ranging from 24% in the Lake Erie basin to 34% in the Lake Superior basin for N. Conversely, about 4.8% (4.1–5.5%) of phosphorus was transported via the groundwater pathway. The proportion of nitrogen load from the septic pathway varies from 2% in the Lake Superior basin (1% for TP) to 4.4% in the Lake Huron basin (2.8% for TP). Controlling much of the landscape nutrient delivery to the US-GLB, Michigan is one of the few states in the US without a statewide septic code governing septic system installation, maintenance, and repair, although discussion and development of such a code is ongoing.^89^

To further investigate the heterogeneity of nutrient delivery, we mapped the amount of TN transported through our four basin transport pathways to streams (overland flow, Figure 7c; tile fields, Figure 7d; groundwater, Figure 7e; septic plume, Figure 7f). Maps for TP transport pathways are shown in Figure S14 because of space limitations and overall similar spatial patterns in TN and TP pathways. For instance, overland transport pathways are high (>238 kg/km^2^/yr TN, >11.97 kg/km^2^/yr TP) in the southern Lake Michigan basin, eastern Lake Ontario basin, and some urban areas (i.e., Detroit, MI; Cleveland, OH; Buffalo, NY; Rochester, NY). The tile field pathway is a major contributor (>1053 kg/km^2^/yr TN, >27.71 kg/km^2^/yr TP) in agricultural areas (e.g., Southern Lake Michigan basin, Saginaw Bay, western Lake Erie, and Lake Ontario basins), likely due to high chemical fertilizer and manure inputs, along with higher tile drainage density. Lakes Erie, Ontario, and southwestern and the thumb area of Lake Michigan showed higher TN and TP delivery through groundwater flow, possibly due to higher groundwater withdrawal rates in these areas and elevated nitrate concentrations.^90^ The septic plume yields were the highest around large cities, where dense suburban populations are not yet connected to sewer systems.

These results also show substantial variability across the US-GLB basins, with different dominant pathways in different Lake basins (Figure 7). Specifically, as the installation of tile drainage expands or intensifies, fluxes from the tile drainage will likely become more important.

TN pathways have higher uncertainties than TP, especially seen in groundwater, overland, and tile field pathways. The standard deviations for these pathways are 5, 3.6, and 2.3% for TN (2.3, 1.4, and 1.5% for TP), respectively. These uncertainties reflect the difficulty of quantifying the nutrient fate and transport, especially through groundwater and tile drainage pathways. Notably, the range of tile fields pathway is from 56 to 70% (nonseptic groundwater pathway: 2–17%) for nitrogen (Table S4) based on the 10% of global optimization runs, while the percentages of the tile field and groundwater pathway from the best-performing parameter set within the local optimization are 46 and 26%, respectively (Figure 6). The slightly different pathway contributions between the best-performing run within the local optimization and ranges from global optimization demonstrated that it is important to conduct nutrient monitoring in the tile-drained water and groundwater systems and that examining seasonal nutrient deliveries across these pathways might be helpful.

## 5 Implications, Limitations, and Future Work

This study uses a spatially explicit nutrient transport model to help us understand the fate and transport of TN and TP from multiple sources along different pathways. Modeling distinct transport pathways provides a novel alternative to many models that do not include important pathways, particularly groundwater and septic plumes. Our analysis shows that overland flow and tile fields are major pathways of nutrient transport, but subsurface transport plays an important role. Specifically, tile drainage is highest in Lake Erie, transporting 44% more TN and 15% more TP than the overland pathway, suggesting that the increasing installation of tile drainage may have significant effects in other regions. For subsurface pathways, groundwater and septic plumes provide 30% of TN delivery and 7% of TP. This will become even more important when we consider legacy nutrients that often have long groundwater travel times.^51,91,92^ Thus, these subsurface pathways should not be ignored in water quality management and policy.

Agricultural nutrient sources (manure, chemical fertilizer, and nitrogen fixation) have played a dominant role in the history of the Great Lakes Basin and will be a critical part of its future. We also found that atmospheric deposition is a significant source of nitrogen and septic tanks contribute significant nitrogen and phosphorus loads to the environment. Groundwater also plays a substantial role in transporting nutrients from the landscape to streams and eventually to the Great Lakes coastline. These findings can be used along with the SENSEflux and SENSEmap products^16^ for the US-GLB to provide managers with spatially explicit loading, efficiency, source, and pathway estimates. For example, the Tipping Point Planner Program links watershed data to local decision-making processes^93^ (https://www.tippingpointplanner.org/); thereby, the addition of SENSEflux can help managers focus actions on specific sources and pathways. The information presented here can provide important inputs to this community-facing tool.

Future research could improve nutrient flux and pathway estimates for the Great Lakes Basin, which would help inform more holistic decisions to achieve nutrient reduction strategies. A more accurate tile drainage map would improve estimates of the contributions of this pathway to the waterways in the basin. In addition, the role of septic plumes in phosphorus delivery and lakes that do not have a connection with streams should be further explored to seek ways that protect water quality by reducing N and P loads. Also, this modeling assumes that all landscape input nutrients have had sufficient time to reach the streams where concentrations are observed and that nutrient inputs are not changing meaningfully over decadal time scales. Future efforts could include time-varied surface loads, along with estimates of legacy time scales and travel times.

## Data Availability

SENSEflux code and model outputs, including nitrogen and phosphorus loads, mass balance components, sources and pathways, along with corresponding watershed summaries at the Hydrologic Unit Code 12 (HUC12) and HUC8 levels published in HydroShare can be found in the MSU Hydrogeology Lab github at: https://github.com/MSUHydrogeology/SENSEflux. Data used in the work are publicly available and are cited in the references. Also, they are accessible through the links below: SENSEmap-USGLB: Nitrogen and Phosphorus Inputs https://www.hydroshare.org/resource/1a116e5460e24177999c7bd6f8292421/.

## Supporting Information Available

SENSEflux model equations (Text S1); spatial distribution of loss terms and basin storage (Text S2); spatial distribution and derivation for in-stream and lake losses (Text S3); ground water recharge and tile-drained area calculation (Text S4); extended model parameter discussion (Text S5); load comparison with the SPARROW model (Text S6); model key inputs (Figure S1); study region and data source (Figure S2); SENSEmap nitrogen and phosphorus sources (Figures S3 & S4) ;spatial load shown nitrogen and phosphorus loads used for model calibration and validation (Figure S5); inputs used to derive river retention factor in SENSEflux (Figure S6); model residual distribution and density (Figure S7 & S8); box plots for SENSEflux model parameter uncertainties (Figure S9); comparison to simulated loads in SPARROW models (Figure S10); TN and TP model loss and attenuation outputs (Figures S11 & S12); estimated percentages of nutrients delivered to lakes by sources (Figure S13); estimated TP yield delivered to lakes by four key pathways (Figure S14); spatial distribution of SENSEflux surface and subsurface partition parameters (Figure S15); optimized model parameters (Table S1); total annual nitrogen and phosphorus flux, yield, and ranges (Table S2); range of source contributions to total basin nutrient delivery (Table S3); and range of pathway contributions to total nitrogen and phosphorus delivery (Table S4) (PDF)
