# Supplementary material for: Important Role of Overland Flows and Tile Field Pathways in Nutrient Transport
Source: Environ Sci Technol. 2023 Oct 23;57(44):17061–75. doi: 10.1021/acs.est.3c03741 (PMC10634344; doi:10.1021/acs.est.3c03741)
Supplement: Supplementary file 1 — es3c03741_si_001.pdf [file es3c03741_si_001.pdf]

Supporting information of

Important Role of Overland Flow and Tile Field Pathways in Nutrient Transport

Luwen Wan*, Anthony D. Kendall, Sherry L. Martin, Quercus F. Hamlin, David W. Hyndman

* Corresponding author: wanluwen@msu.edu

Author Information:

Luwen Wan - Department of Earth and Environmental Sciences, Michigan State University, East Lansing, MI, 48824, United States; **Present Address**: Department of Earth System Science, Stanford University, Stanford, CA, 94305, United States; Institute for Human-Centered Artificial Intelligence, Stanford University, Stanford, CA, 94305, United States.

Anthony D. Kendall - Department of Earth and Environmental Sciences, Michigan State University, East Lansing, MI, 48824, United States.

Sherry L. Martin - Department of Earth and Environmental Sciences, Michigan State University, East Lansing, MI, 48824, United States; **Present Address**: USGS Upper Midwest Water Science Center, Lansing, MI, 48911, United States.

Quercus F. Hamlin - Department of Earth and Environmental Sciences, Michigan State University, East Lansing, MI, 48824, United States; **Present Address**: Airspace Link, Inc. 2050 15th Street, Detroit, MI 48216, Suite 3-100, Detroit, MI 48226, United States.

David W. Hyndman - Department of Earth and Environmental Sciences, Michigan State University, East Lansing, MI, 48824, United States; **Present Address**: Department of Geosciences, School of Natural Sciences and Mathematics, The University of Texas at Dallas, Richardson, Texas 75080, United States.

Contents of this file

(1) 6 Texts (pages S3 – S9)

Text S1. SENSEflux model equations.

Text S2. Spatial distribution of loss terms and basin storage.

Text S3. Spatial distribution and derivation for in-stream and lake losses.

Text S4. Groundwater recharge and tile-drained area calculation.

Text S5. Extended model parameter discussion.

Text S6. Load comparison with the SPARROW model.

(2) 15 Figures (pages S9 – S22)

Figure S1. Model key inputs.

Figure S2. Study region and data source.

Figure S3. SENSEmap nitrogen source for US-GLB.

Figure S4. SENSEmap phosphorus source for US-GLB.

Figure S5. Spatial domain showing nitrogen and phosphorus loads used for model calibration and validation.

Figure S6. Inputs are used to derive the river retention factor in SENSEflux.

Figure S7. Model residual ($\log_{10}$ model – $\log_{10}$ observed) distribution and density.

Figure S8. Log model residuals (kg/day) by watersheds for both calibration and validation datasets.

Figure S9. Box plots of SENSEflux model parameter uncertainties.

Figure S10. Comparison to the simulated loads in the SPARROW models.

Figure S11. TN model loss and attenuation outputs: harvest, basin loss, river, and lake attenuation.

Figure S12. TP model loss and attenuation outputs: harvest, storage, basin loss, river, and lake attenuation.

Figure S13. Estimated percentages of total nutrients delivered to lakes by sources.

Figure S14. The estimated TP yield is delivered to lakes by four key pathways.

Figure S15. Spatial distribution of SENSEflux surface and subsurface partition parameter.

(3) 4 Tables (pages S23 – S24)

Table S1. Summary of optimized model parameters.

Table S2. Total annual nitrogen and phosphorus flux and yield and ranges.

Table S3. Range of source contributions to total basin nutrient delivery.

Table S4. Range of pathway contributions to total nitrogen and phosphorus delivery.

(4) Reference (pages S25 – S26)

S1. SENSEflux model equations

The main function of SENSEflux modeling is shown in equation (1) below. Where L_k is the simulated load (kg/day) at the sampling location for an individual catchment. S represents the nutrient source, S_{point} is the point source and directly applied into the rivers and streams, S_{sep} is the septic tank source, and S_{ij} is the application of other sources i that can be harvested to watershed cell j. $SepEff$ is removal efficiency on septic loads, it's fixed as 0.3 for total nitrogen and 0.35 for total phosphorus[1,2]. ExH_{ij} is an extraction factor that describes the in-place root zone removal of nutrients before transport (such as Harvest). For all other cells and sources, ExH_{ij} is equal to 1. F_j is a subsurface partition parameter, describing the fraction of nutrients that are transported via a subsurface pathway. It is a function of normalized groundwater recharge fraction (recharge fraction in a cell j divides the maximum recharge fraction across the SENSEflux model domain), where $rechF_j$ is the groundwater recharge fraction in cell j, and $max(rechF)$ is the maximum value of groundwater recharge fraction across the SENSEflux model domain (equation (2)). Recharge fraction was defined as the average annual precipitation becoming recharged and is limited by 0.55 across the basin[3].

$$L_k = \sum_{j}^{cells} R_j Lacus_j \times \{S_{point} + S_{sep}(1 - SepEff)Bse_j + \sum_{i}^{\substack{other\\sources}} S_{ij} \times ExH_{ij} \times \left[\left(1 - F_j\right)Bs_j + F_j\left(1 - Fstor_{ij}\right)Bg_j\right]\} \quad (1)$$

$$F_j = f \times \left(\frac{rechF_j}{\max{(rechF)}}\right) \quad (2)$$

A major update between SENSEflux and its former version[1] is an updated river retention function (equation (3)-(6)), lake retention, and subsurface phosphorus storage with two new model parameters $Lacus_j$ and $Fstor_j$, defined in Equation (7) and (8) respectively. R_j describes river reduction of the remaining nutrients after landscape attenuation, and it split as denitrification for TN (sorption for TP) as well as biological uptake & burial (equation (3)). For N denitrification or P sorption (equation (4)), it is an exponential function of $DNSP_j$ that was calculated based on the flow length tool and used streambed interaction as an input weight raster. The streambed interaction rate calculation is shown in equation (6), where $\hat{k}$, $\hat{s}$, and $\hat{b}$ are derived from hydraulic conductivity, slope, and basin yield respectively, R and V represent hydraulic radius and velocity, see details in Supporting Information (S3). Biological uptake & burial is an exponential function of T_j that represents in-stream travel time from cell j to the downstream observation point (equation (5)).

$$R_j = e^{-\alpha * DNSP_j} * \ e^{-\alpha 1 * Bio_j} \tag{3}$$

$$DNSP_j = flowLen(streambedInteraction_j) \tag{4}$$

$$Bio_j = \ flowLen(inStreamTravelTime_j) \tag{5}$$

$$streambedInteraction_j = \frac{\hat{k}_j * \hat{s}_j * \left(1 - \hat{b}_j\right)}{HR_j * V_j} \tag{6}$$

We also consider nutrient attenuation via lakes or reservoirs (*Lacus$_j$*) as they travel down the stream network, which is a function of travel distance in lakes (equation (7)), and only the loss for a lake or reservoir which has a connection with streams is considered due to the inherent river routing scheme in the SENSEflux model. *Fstor$_j$* is the fraction of groundwater pathway nutrients stored/lost in the soil and the deeper unsaturated zone where *fstor* is a calibrated constant (equation (8)). *fstor* was assumed to be zero due to the high mobility of nitrogen. By adding this term, we can estimate the amount of phosphorus in every grid cell delivered to the streams.

$$Lacus_j = e^{-\alpha 2 * DL_j} \tag{7}$$

$$Fstor_j = fstor \times \left(1 - \frac{rechF_j}{\max\,(rechF)}\right) \tag{8}$$

All basin attenuations are exponential functions of flow length (D) from each cell to the nearest downgradient stream cell. *Bo$_j$*, *Bg$_j$,* and *Bs$_j$* are basin reduction parameters, representing overland flow, non-septic groundwater, and septic plume pathways respectively (equations (9), (11), (12)). Tile field pathway *Bt$_j$* is also considered in the model as an alternative overland pathway, representing nutrient attenuation along with tile fields if tile exists in a cell (equation (10)). Equations describing each of these terms are given in Luscz et al (2017)[1] and Martin et al (2021)[4].

$$Bo_j = e^{-bo * D_j} \tag{9}$$

$$Bt_j = e^{-bt * D_j} \tag{10}$$

$$Bg_j = e^{-bg * D_j} \tag{11}$$

$$Bs_j = e^{-bs * D_j} \tag{12}$$

S2. Spatial distribution of loss terms and basin storage

Three in-situ loss terms are applied before nutrients are transported: Septic removal (*SepEff*), Harvest (*ExH*), and Subsurface storage (*Fstor*). Septic removal efficiency is applied on the septic load and fixed as 0.3 for N and 0.35 for P [1,2]. Harvest includes all in-place root zone nutrient loss and is assumed to occur in cells with manure or chemical agricultural fertilizers applied. The Subsurface storage loss term includes both in-place storage and loss of nutrients below the root zone, which we assume to occur for phosphorus (Eq (8)). *Fstor* was assumed as zero for nitrogen due to the high mobility of N. By adding this term, we can estimate the number of nutrients in every grid cell delivered to the streams.

S3. Spatial distribution and derivation for instream and lake losses

The attenuation of nutrients during stream/wetland transport is assumed to be broken into two components: 1) water column and sediment interface losses, and 2) hyporheic zone losses. Water column losses consist of biological uptake, followed by subsequent denitrification and particulate transport. For phosphorus, sediment burial is another active process. Hyporheic zone losses may be biological uptake (N or P), denitrification (N), sorption (P), or mineralization (P). We label the corresponding terms “water column (WC)” and “hyporheic zone (HZ)” attenuation.

Water column attenuation is assumed to be a function of travel time in the stream/wetland, given as $Loss_{wc} = \alpha_{wc} T_s$, where α_{wc} is a calibrated constant across the domain, and T_s is the travel time in-stream. Travel time in-stream in each model cell is given by $T_s = \frac{1}{v}$ where v is the velocity of water in that stream cell. While this is the loss in a single cell, integrating across all cells along the flow path results in the delivery of nutrients $N_{out} = N_{in} R_{wc}$, where $R_{WC} = e^{-\alpha_{wc} TT_s}$. Here, TT_s is the total travel time in streams, computed in ArcGIS as described in the main text (Section 3.5).

To compute losses due to streambed and hyporheic zone interactions beneath it, we define losses in this zone to be given by the residence time multiplied by a static constant, $Loss_{hz} = \alpha_{hz} \tau_{hz}$ where τ_{hz} is the residence time in the hyporheic zone, defined as $\tau_{hz} = D/v_{hz}$ where D is the hyporheic zone depth and v_{hz} is the velocity of water flowing into/out of the HZ.

The streambed interaction factor (D) is given by $D \sim f(K, S, 1/Rech)$, where K is the hydraulic conductivity of the streambed sediments, S is the slope of the stream channel, and $Rech$ is groundwater recharge. Thus, we have assumed that higher streambed sediments increase the exchange of surface and groundwater (increasing hyporheic zone depth) and that a higher slope leads to greater streambed morphometric variability, and thus greater incidence of flow paths entering the sediments and exiting shortly thereafter, promoting hyporheic exchange. We further assume that greater groundwater recharge upstream leads to a stronger influx of water from below, which would reduce the depth of exchange through the groundwater pushing back against streamflow. Here, recharge is assumed to be represented by the basin yield of streams at their 30th percentile, thus $R \sim BY_{30}$. We chose to normalize each of the terms by their maximum and minimum values across the model domain, and we log-transformed K. The final equation for hyporheic zone depth D is:

$$D = \alpha_D \cdot \left[\frac{\log\left(\frac{K}{K_{min}}\right)}{\log\left(\frac{K_{max}}{K_{min}}\right)} \right] \cdot \left[\frac{S}{S_{90}} \right] \cdot \left[1 - \frac{BY_{30}}{BY_{30,max}} \right] = \alpha_D \widetilde{K} \cdot \tilde{S} \cdot \widetilde{BY} \quad (13)$$

Where each of the terms with a tilde (~) represents 0-1 normalized values, corresponding to the three-square bracket sections of the left-hand side of the equation. The unit of D is [L] (here, m). Note for $\tilde{S}$ we limited the maximum value to 1.

If we assume that the total flux of nutrients into the HZ (n_{in}) is given by $n_{in} = Q_{hz} C_{in}$, where Q is the flux of water in/out of the HZ [L^3/T] and C_{in} is the input concentration to the HZ [M/L^3], we can represent $Q_{hz} = P \cdot 1 \cdot v_{hz}$, where P is the perimeter of the stream channel [L], 1 is the unit length of the channel [L], and v_{hz} is the velocity of water exchange in the HZ [L/T]. Therefore,

$n_{in} = P \cdot v_{hz} \cdot C_{in} = \frac{P \cdot v_{hz} \cdot N_{in}}{Q}$, where N_{in} is the input nutrient flux [M/T] in the stream channel above the HZ, and Q is the streamflow in the channel [L^3/T].

We further assume that nutrient uptake is linearly related to the residence time in the HZ, τ_{hz} which can be expressed as $\tau_{hz} = D/v_{hz}$. Thus, the flux of nutrients out $n_{out} = n_{in} \cdot \tau_{hz} \cdot \alpha_{hz}$, where α_{hz} is a parameter to be calibrated in the model. Substituting the definitions of n_{in} and τ_{hz}, we get $n_{out} = \frac{P \cdot v_{hz} \cdot N_{in}}{Q} \cdot \frac{D}{v_{hz}} \cdot \alpha_{hz}$. Canceling terms, we get $n_{out} = \frac{P}{Q} \cdot N_{in} \cdot D \cdot \alpha_{hz}$. We can further use the relationship between P (the wetted perimeter of the stream channel) and $Q = A \cdot v$ [L^3/T], determined by the hydraulic radius $R = A/P$ [L], where A is the channel area, and v is the in-stream average water velocity. Thus $\frac{P}{Q} = \frac{A}{R \cdot A \cdot v} = \frac{1}{R \cdot v}$. Finally, if we substitute this and equation (13) into our equation for n_{out} (noting that the linear constants combine $\alpha_{hz} = \alpha_{hz} \cdot \alpha_D$) we get the equation (14).

$$n_{out} = N_{in} \frac{\boldsymbol{\widetilde{K} \cdot \tilde{S} \cdot \widetilde{BY}}}{\boldsymbol{R \cdot v}} \alpha_{hz} \tag{14}$$

The bold terms in Equation (14) are independent of nutrient concentrations but vary in space. Therefore, we combine these terms into a single model input layer we term the streambed exchange rate, $SE = \frac{\widetilde{K} \cdot \tilde{S} \cdot \widetilde{BY}}{R \cdot v}$ [T/L], shown in Figure S6e. Other intermediate inputs to calculate SE are shown in Figures S2 and S6. While the values of K and S could be calculated from input static layers (e.g., Figure S2d and Figure S6a), BY, R, and v needed to be computed for each point along the stream channel. For this, we used the at-many stations hydraulic geometry method[5], deriving the functional power-law relationships that relate median velocity, and hydraulic radius to discharge. More specifically, we first extracted river geometries, including discharge, velocity, width, and area, from USGS gauges across the basin from 1/1/2000 to 12/31/2019. Hydraulic radius was then calculated as area divided by width with the assumption that river channels are rectangular. Then, the log-linear fit relationship was built for stream geometries as a function of median discharge for all gauges within the period. 75% of the measurements are used to fit the model and the remaining 25% are used to validate the model, r squared values are used to assess the fitting performance. Lastly, we created a stream geometry raster for the USGLB with the following steps. First, calculation of basin yield. During this step, calculated flow accumulation based on DEM (Section 3.5) is extracted for all the USGS gauges, then up to 20% of the difference between the flow accumulation and reported area is allowed, quality assurance/quality control (QA/QC) are performed to remove outliers afterward, and finally nibble tool was used to replace cells with missing values by the value of their nearest neighbor. We then calculated streamflow for every cell by multiplying the nibbled basin yield with flow accumulation. Then, in-stream velocity and hydraulic radius across the basin were calculated based on the streamflow and derived relationships at gauges between discharge and stream geometries.

S4. Groundwater recharge and tile-drained area calculation

Groundwater recharge was estimated based on a series of linear models derived from the Landscape Hydrology Model (LHM), a coupled process-based hydrological model[3]. LHM was originally developed for the Muskegon River Watershed, located in the central part of Michigan's lower peninsula, and contains diverse land use representative of the broader region. This hydrological model combined several GIS layers including land use, soils, and station observation data to predict stream discharge, groundwater recharge, and evapotranspiration from 1990 to 2004. The linear regression models fit each land use type to the percentage of precipitation that becomes recharge as a function of soil hydraulic conductivity. The annual precipitation from 2008 to 2012 was downloaded from Parameter-elevation Regressions on Independent Slopes Model (PRISM) database[6] and average annual precipitation from 2008 to 2012 was used for linear models. The soil hydraulic conductivity is derived from the soil texture of the soil survey geographic database (SSURGO)[7] and land cover data from the national land cover database 2011[8]. The recharge estimates for US-GLB are shown in Figure S2.

To derive an estimated tile drainage map, we used GIS-based mapping based on the premise that crops grown on land with low slopes and poorly drained soil likely have tile drains. First, cells classified in the 2011 NLCD[8] as "Cultivated Crops" were extracted. Then, we fit a model to land drained by tile county-level data from land-use practice in 2012 for the 109 counties in the US-GLB (Figure S6), published by the United States Department of Agriculture, National Agricultural Statistical Service[9]. 55 counties were randomly selected as training datasets and the remaining 54 counties as validation datasets. This model selects areas that are cultivated land-use types, moderately low soil permeability (Ksat < 14.4 mm/hr), and low average slopes (< 1.2%) as tile drainage (r^2 = 0.83). The rest of the 54 counties were used to calibrate the model (r^2 = 0.85). Estimates of tile-drained areas are shown in Figure S6. The aggregation method (maximum) used in pre-processing data may overestimate the area of tiled fields in the model.

S5. Model parameter extended discussion.

Four of the model parameters (*f, ExH, SepEff, and fstor*) are linear coefficients on loss terms in the model, while the remainder (basin and river losses) are coefficients in the exponent of an attenuation term—thus their values are not directly comparable.

The subsurface partition parameter f is multiplied by the normalized recharge (equation (2)) and represents the proportion of mobile surface-applied nutrients (after Harvest) that are sent through the groundwater pathway. Thus, areas with the highest recharge in the basin have 49% of mobile surface-applied nutrients sent to groundwater for N while 78% is routed through groundwater for P (Table S1). See Figure S14 for a map of the final groundwater partition fraction. It is somewhat surprising that phosphorus has a higher fraction than nitrogen, given the relative ease with which nitrate in particular leaches from soils[10], however, this may be an artifact of the simplistic relationship between recharge rates and groundwater pathway hydrologic fractionation imposed here. It may also be due to a non-linear relationship between soil texture (underlying recharge) and P mobility. P moves much more readily through sandy soils than finer textured ones[11]. That relationship is also not captured properly here.

Our model predicts that 80% of surface-applied N is harvested, lost to the atmosphere (N only), or stored in the root zone in agricultural settings, while 95% of P is. Figures S10a and S11a show total losses due to harvest, atmosphere, and storage for N and P, respectively. The higher rate of P harvest may be influenced by the tendency of P to sorb to unsaturated zone soils, rather than a more careful accounting of N and P needs when fertilizers are applied.

Phosphorus then experiences an additional deep unsaturated zone storage, where up to 55% of groundwater-mobile P (in the lowest recharge areas, Eq (8)) is stored (Figure S11b). Areas with higher recharge then experience less storage proportionately.

Nutrient attenuation during basin transport through surface runoff, tile drain fields, general groundwater, and septic plumes are determined by the overland travel distance along with the corresponding parameters (*bo, bt, bg, and bs,* equations (9) – (12)). The lower calibrated parameter value means a higher delivery rate through the pathway, but the number of nutrients delivered through these pathways are not directly comparable due to the different amounts traveled before basin attenuation. Overland flow and tile drains are dominant pathways (Table S1) while groundwater and septic plumes have relatively fewer deliveries. Nutrient losses during nutrient transport across the landscape are determined by travel distance and the calibrated basin reduction parameters (Table S1), and the losses are shown in Figure S10b (TN) and Figure S11c (TP). P attenuations are mainly concentrated in the southern basin with agricultural soils, while N losses have more spatial variability.

Within stream and lake attenuation, water column losses (*bio*) are higher than streambed and hyporheic zone losses (*dnsp*) for both TN and TP (Table S1). The losses for TN and TP are shown in Figure S10c and Figure S11d, respectively. Generally, Areas close to the coastline have less stream and lake attenuation for TP due to less travel time.

S6. Load comparison with the SPARROW model

First of all, there are some important differences to note: SPARROW does not use spatially explicit nutrient sources nor attenuation processes and is run at a coarser resolution. Using the same observation dataset, the SENSEflux TN model slightly underestimated high loads while the SPARROW model slightly overpredicted them (Figure S10). Both SENSEflux and SPARROW models slightly underpredicted higher TP loads and overpredicted lower loads. These differences are not surprising because the two models have several notable differences, including nutrient attenuation processes, methods to model nutrient sources, spatial resolution, and timeframes. Specifically, SENSEflux includes four distinct pathways (tile fields, overland, septic plumes, and groundwater, see Figure 1) while the SPARROW model uses data on land-to-water delivery factors, such as soil permeability, drainage density, precipitation, air temperature, the fraction of the stream catchment with tile drains to describe attenuation processes broadly across the basin[12]. Moreover, SENSEflux uses spatially explicit nutrient source inputs from SENSEmap while SPARROW uses land use/cover and county-level estimates of nutrient masses to statistically compute sources more generally[13]. Finally, SPARROW models were developed for TN and TP

with a 2002 base year[12], while nutrient sources and watershed factor data used in SENSEflux are based on ca. 2010 data.

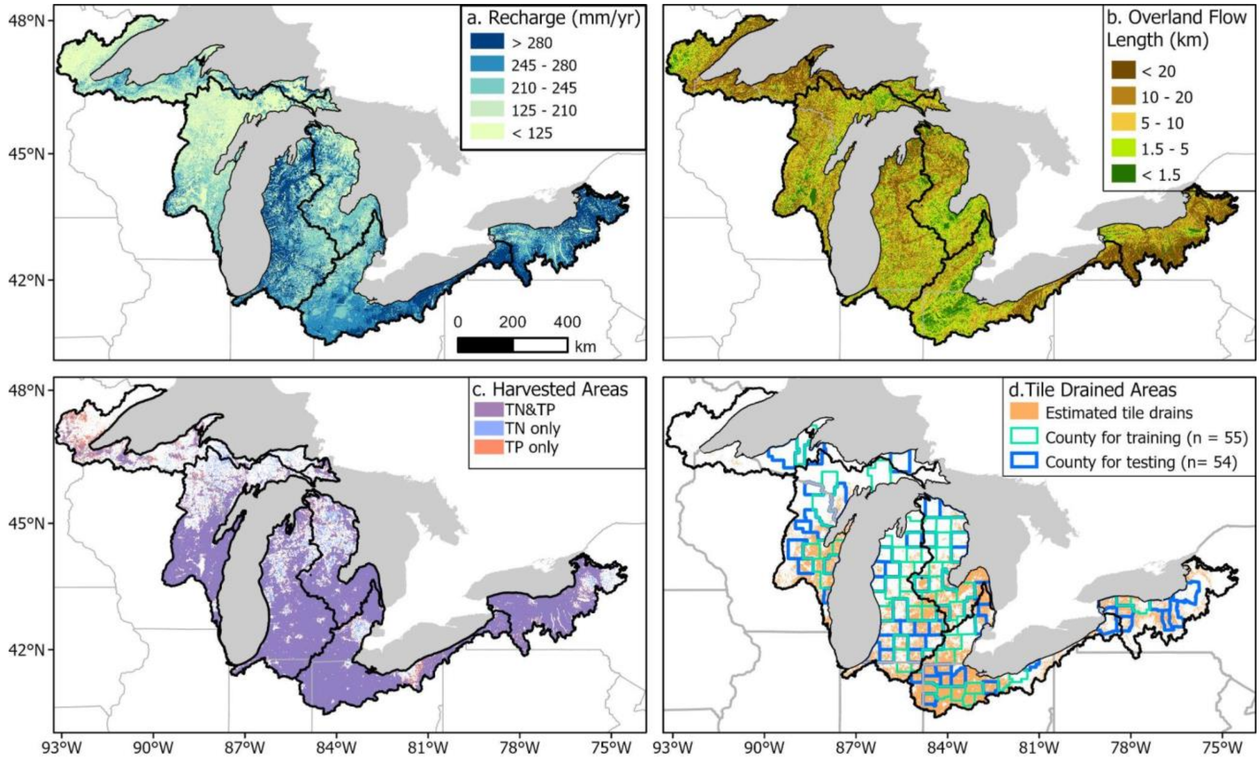

Figure S1. Model key inputs. (a) annual groundwater recharge for US-GLB; (b) overland flowlength; (c) harvested areas where either manure or chemical agricultural fertilizers applied for TN and TP; (d) estimated tile drainage (indicated in yellow) estimated from land use, soil permeability, and average slope

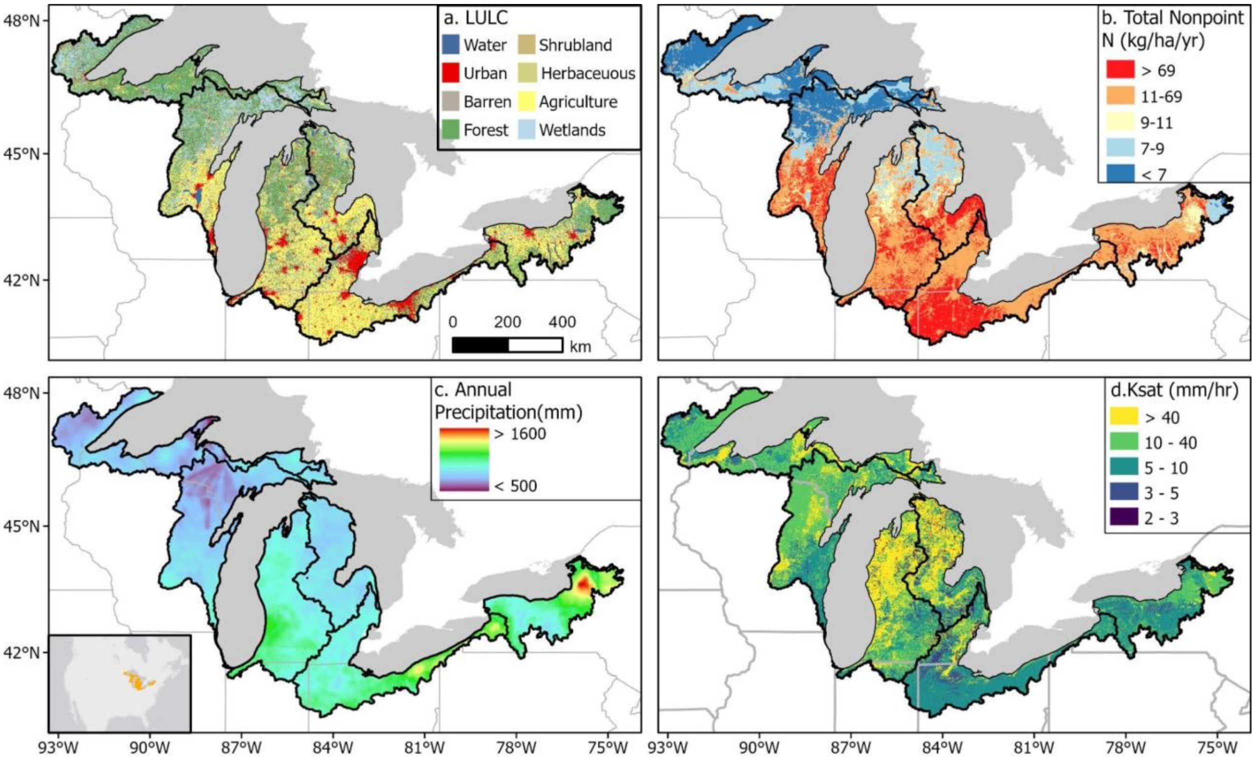

Figure S2. Study region and data source showing the GLB in the inset map, along with a. the Land Use/Land Cover across the basin from the National Land Cover Dataset (USGS, 2011); b. non-point TN source from SENSEmap (Hamlin et al., 2020a, b); c. Average annual precipitation from 2008 to 2012 from PRISM (PRISM Climate Group 2011); d. the saturated conductivity of the top soil layer from SSURGO (Soil Survey Staff, 2022).

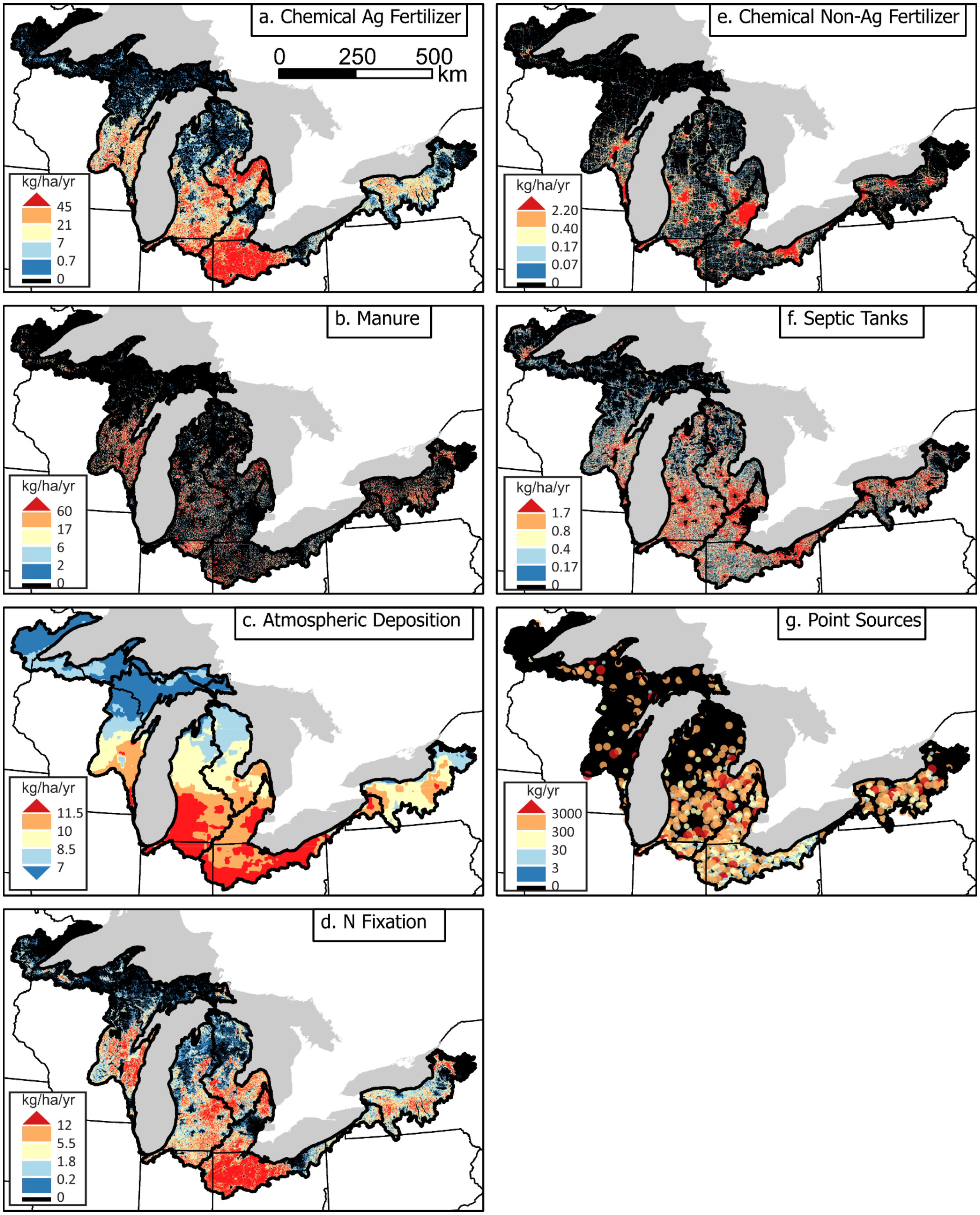

Figure S3: SENSEmap nitrogen source for US-GLB resampled to 720 m resolution for display, derived from Hamlin et al (2020). The units are kg/ha/yr except for point sources (kg/yr). The color breaks are based on quantile classification and round-off methods.

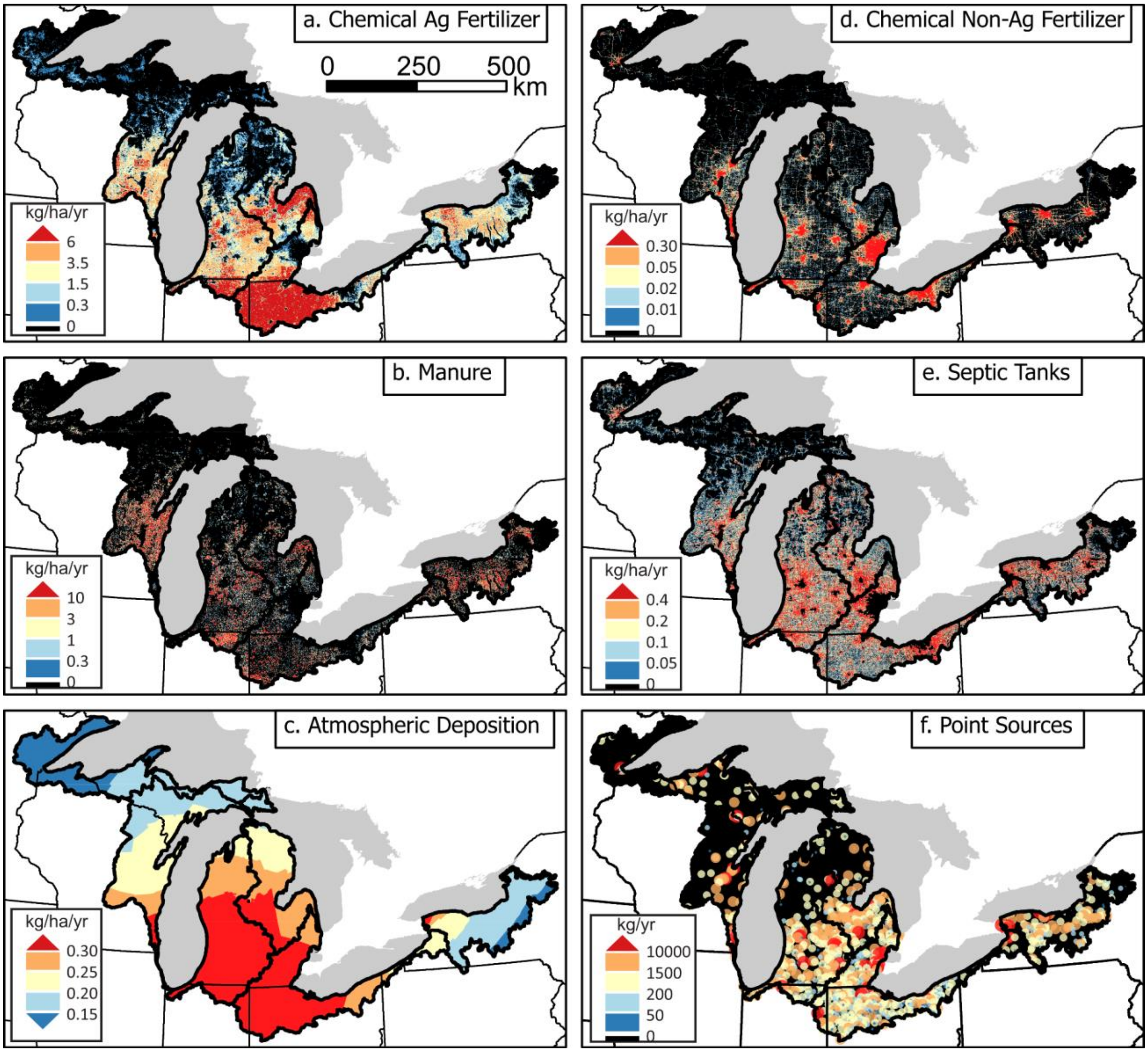

Figure S4: SENSEmap phosphorus source for US-GLB resampled to 720 m resolution for the display. The units are kg/ha/yr except for point sources (kg/yr). The color breaks are based on quantile classification and round-off methods.

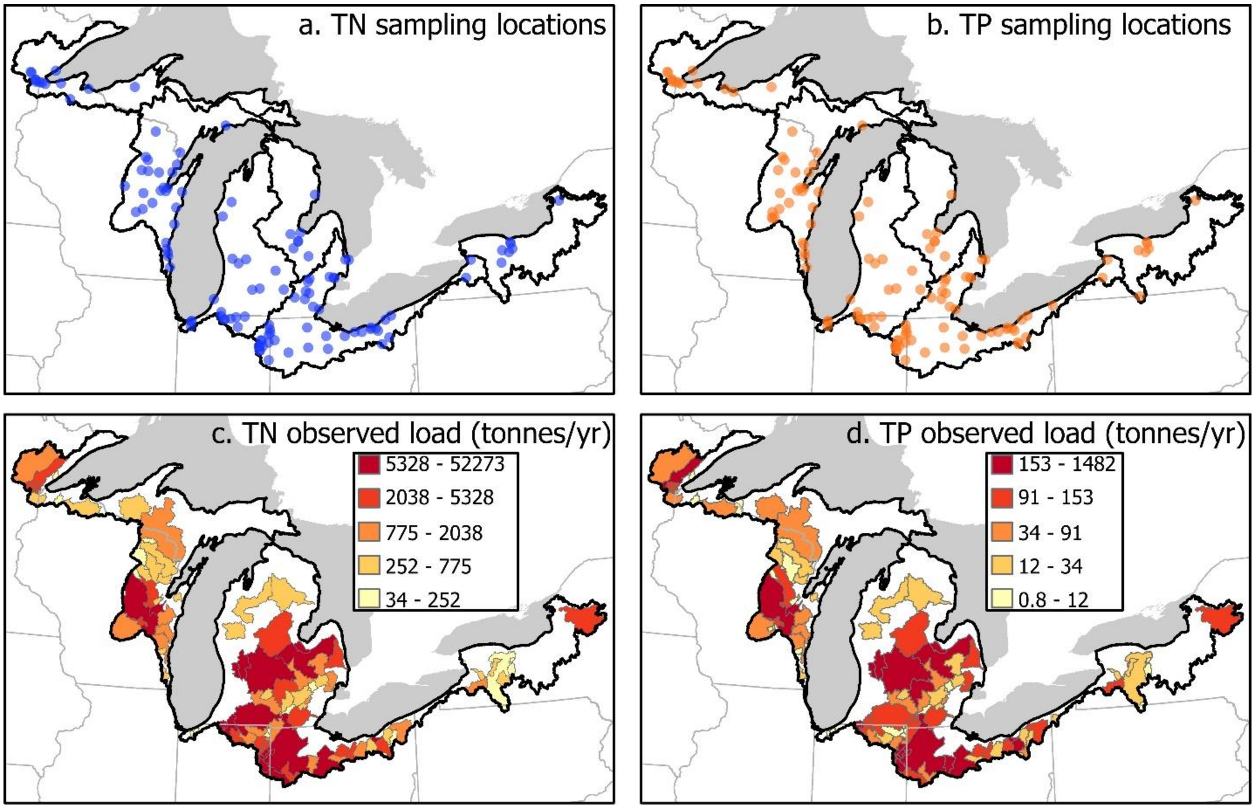

Figure S5. Spatial domain showing nitrogen and phosphorus sampling locations that are used for delineating watersheds and loads used for model calibration and validation. (a) TN sampling locations (N = 116), (b) TP sampling locations (N = 119), (c) TN watersheds with loading, and (d) TP watersheds with loading. Maps are classified in quantiles, with each color representing 25% of the study domain.

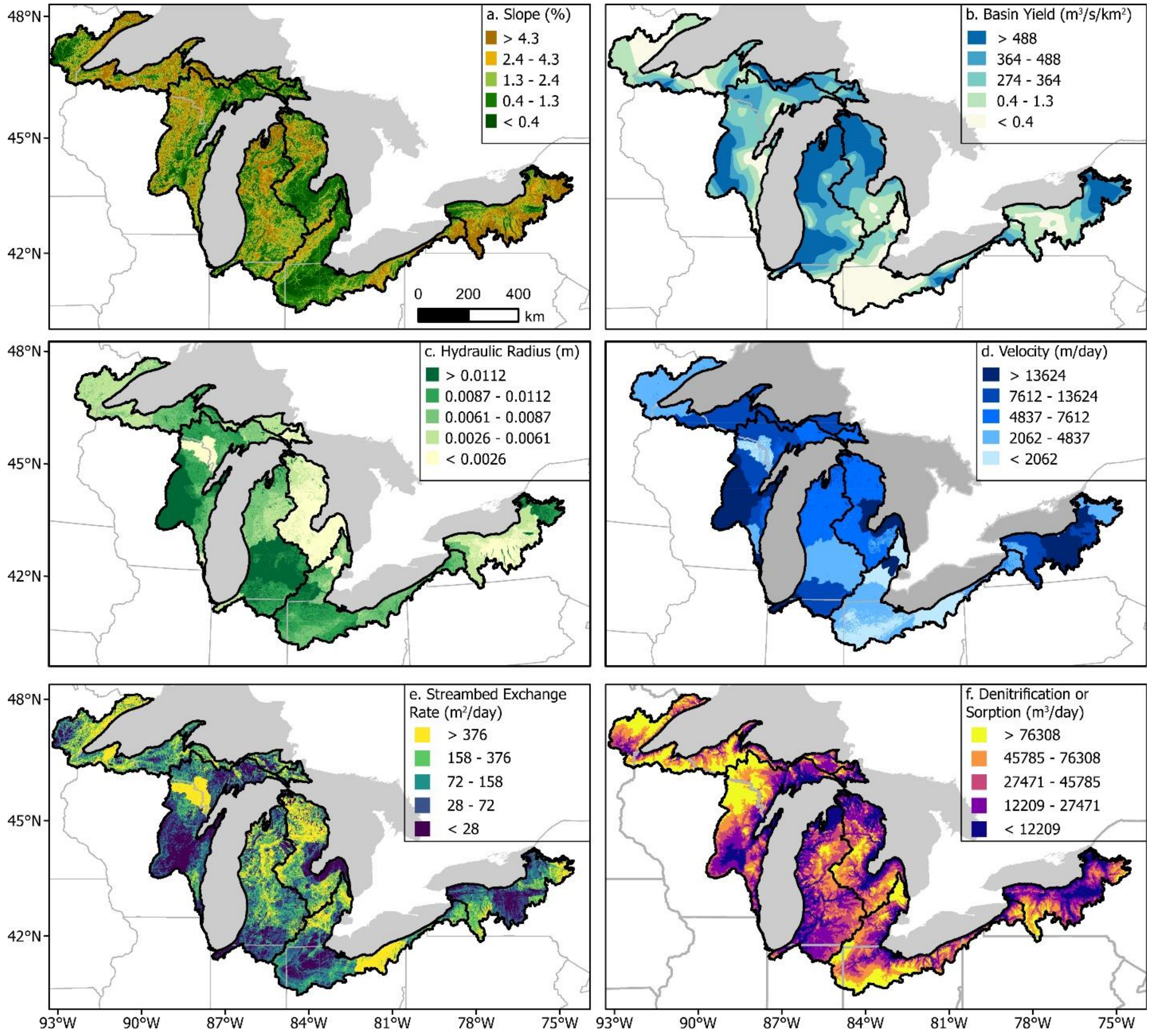

Figure S6. Inputs are used to derive the river retention factor in SENSEflux. (a) average slope, (b) basin yield during baseflow; (c) hydraulic radius; (d) average velocity; (e) streambed exchange rate; (f) N denitrification or P sorption factor.

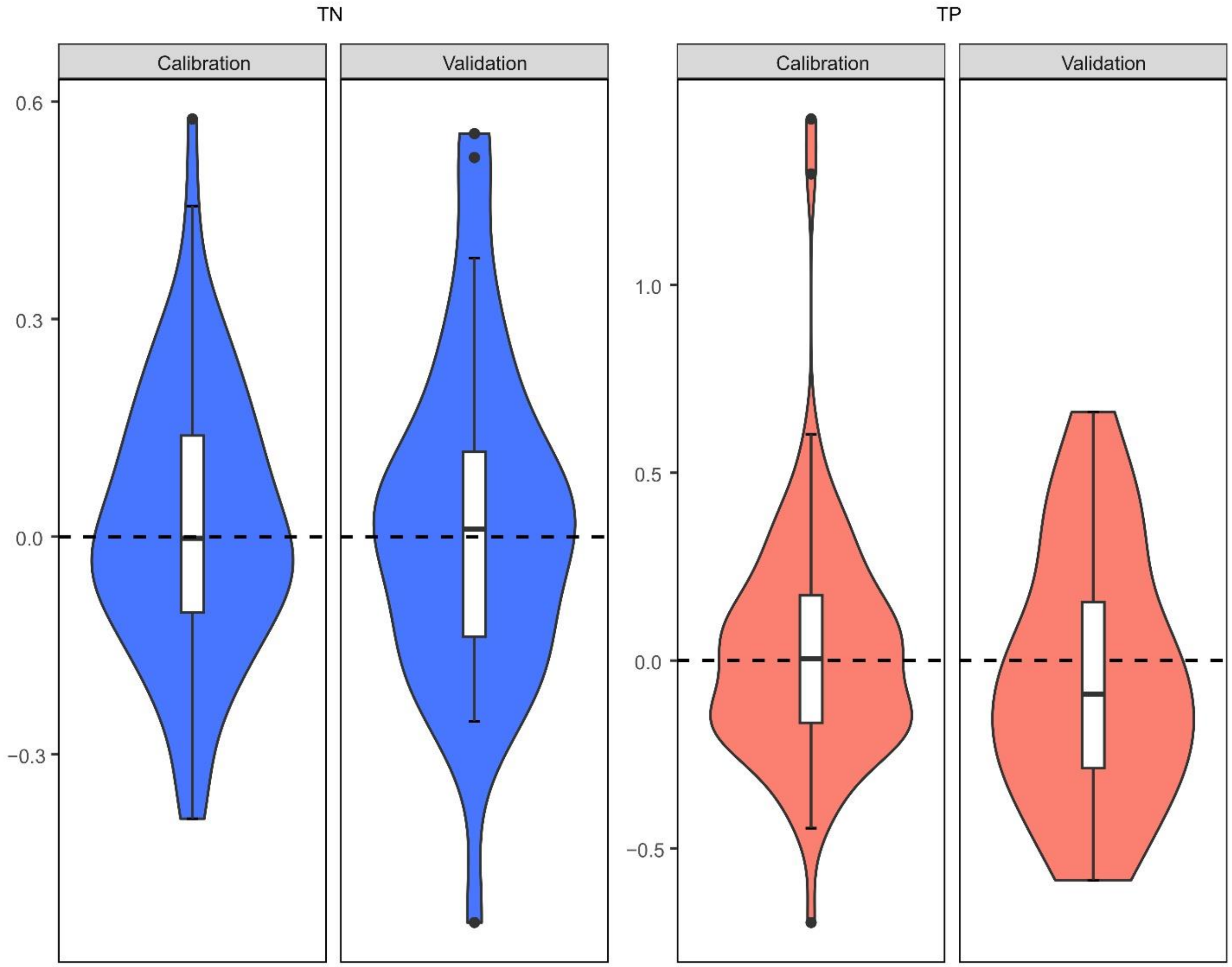

Figure S7. Model residual ($\log_{10}$ model – $\log_{10}$ observed) distribution and density are shown as violin plots. The bottom and top of each box represent the first and third quartiles, respectively, and the line inside each box represents the median. Zero residual is indicated as a black dashed line. The top and bottom bars (whiskers) represent the maximum and minimum residuals, respectively. Data beyond the end of the whiskers are "outlying" points and are plotted individually. None of the means were significantly different from zero, as measured by the one-sample t-test, with P values > 0.05.

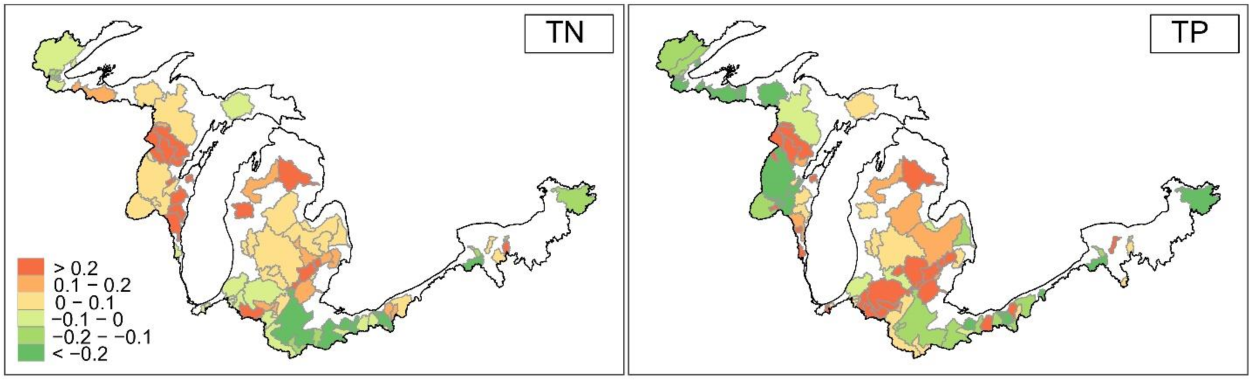

Figure S8. Log model residuals (kg/day) by watersheds for both calibration and validation datasets. The color breaks are based on quantile classification and are rounded to the nearest 0.1.

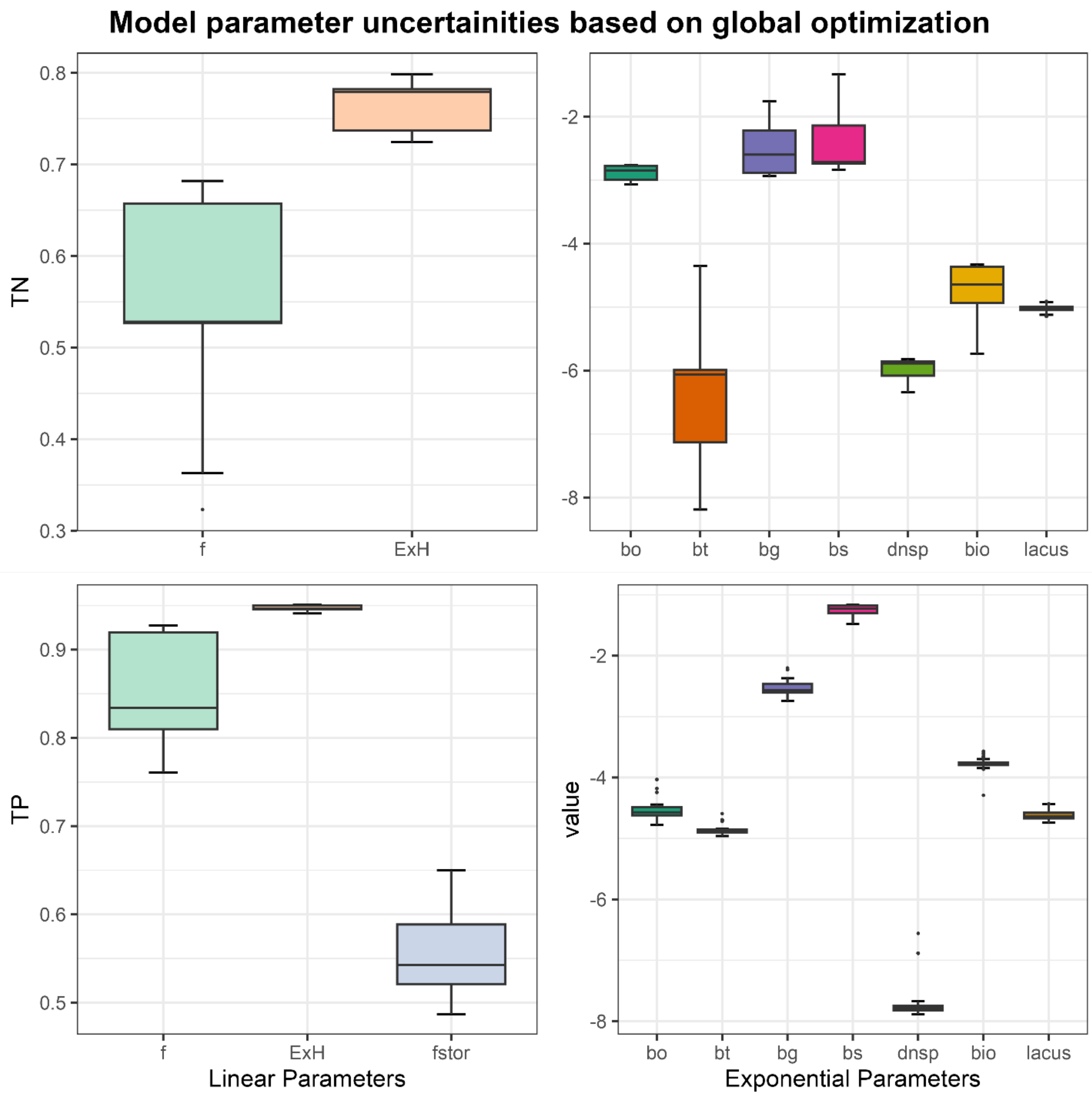

Figure S9. The boxplot represents the median, 25th and 75th percentile and max value within 1.5 times the interquartile range above the 75th percentile, and minimum value within 1.5 times the interquartile range below the 25th percentile. These values are based on 10% of global optimization runs with lower objective function (MAEL) values (100 for TN, 78 for TP).

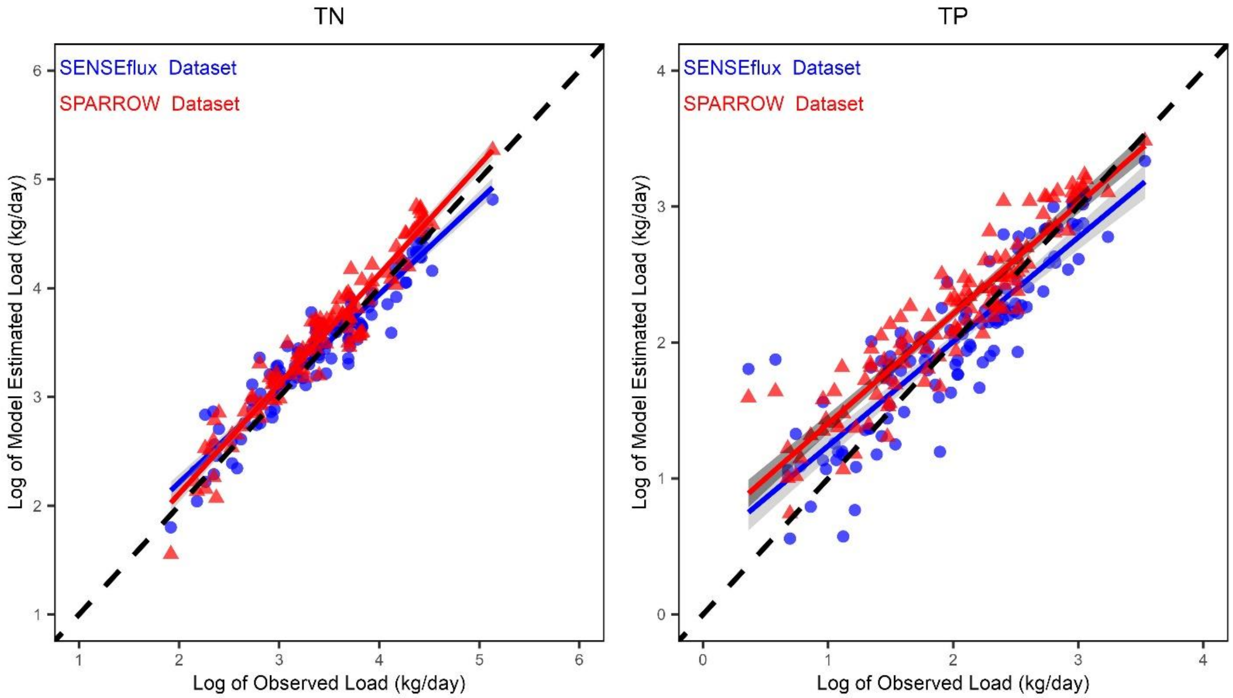

Figure S10. Comparison to the simulated loads ($\log_{10}$ of kg/day) in the SPARROW models. Blue dots and lines are for SENSEflux simulation, and red is for SPARROW. The dashed black line is a 1:1 line, where simulated loads are equal to observed loads.

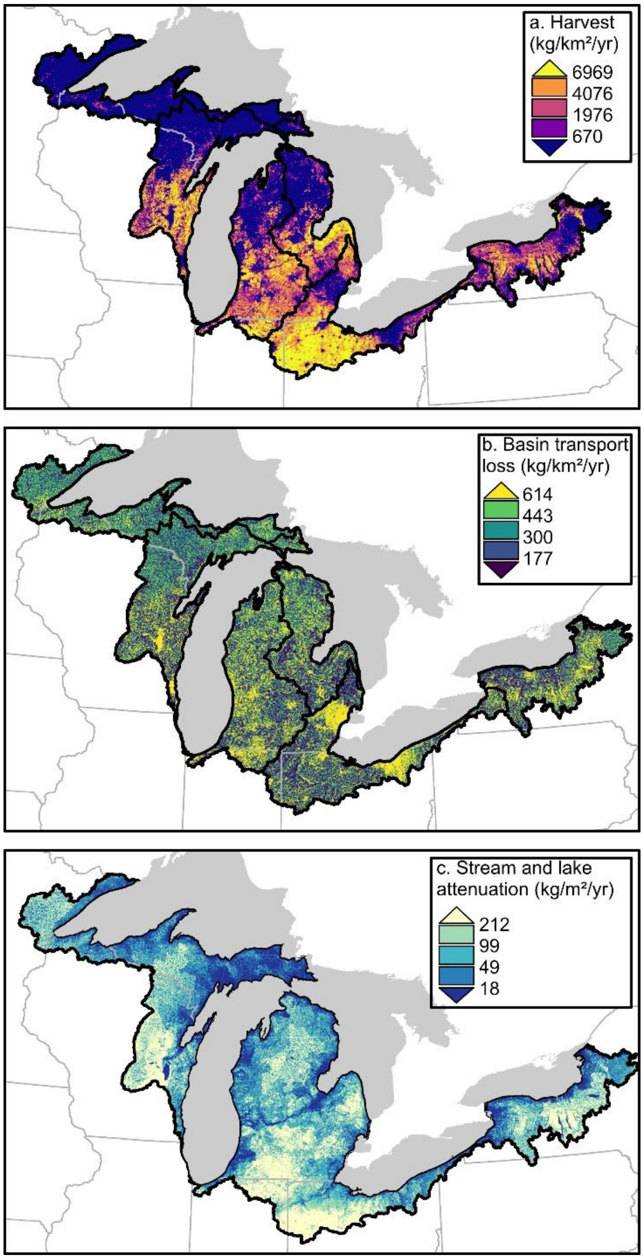

Figure S11. TN model loss and attenuation outputs. (a) crop extraction of nitrogen; (b) total nitrogen loss during basin transport; (c) nitrogen uptake in streams and connected lakes. Maps are resampled from 120m SENSEflux outputs to 720m resolution here for display purposes and classified in quantiles with each color representing 20% of the dataset.

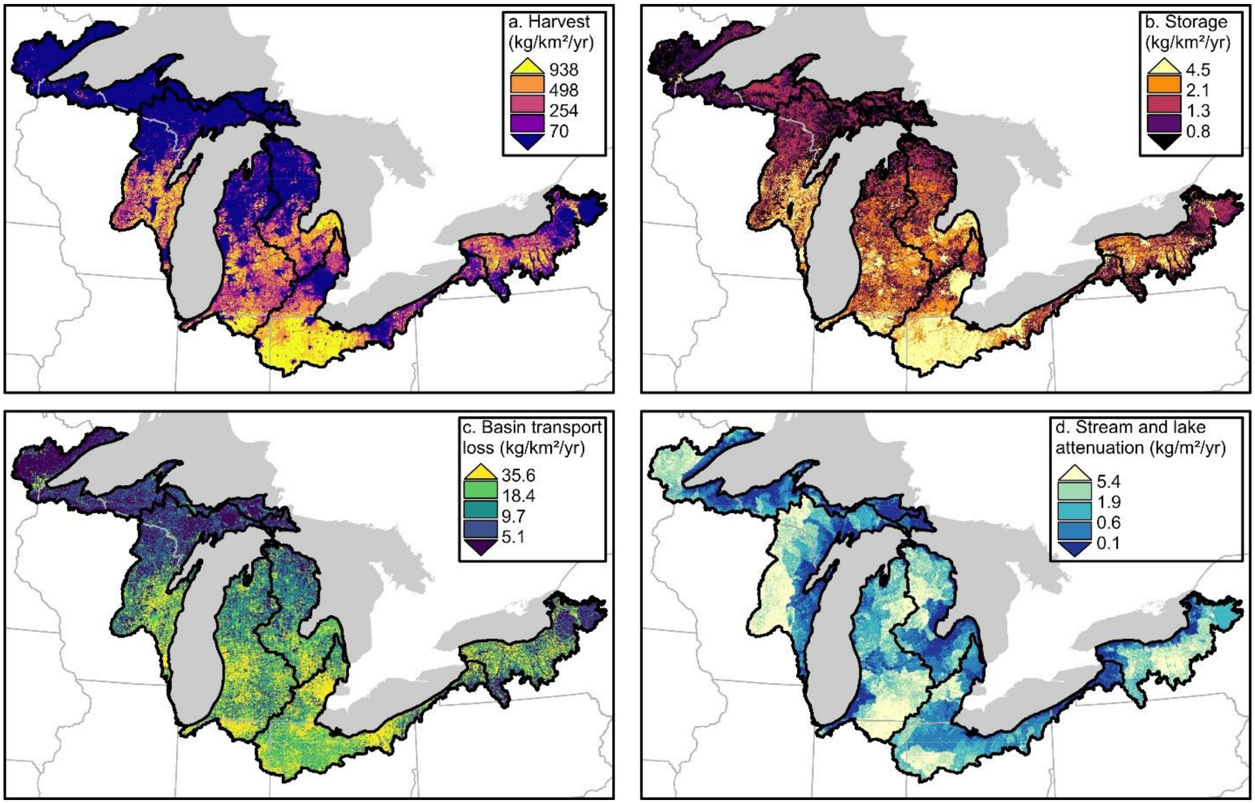

Figure S12. TP model loss and attenuation outputs. (a) crop extraction of phosphorus; (b) in-place phosphorus storage and loss of phosphorus below the root zone; (c) TP loss during basin transport; (d) phosphorus uptake in streams and connected lakes. Maps are resampled from 120m SENSEflux outputs to 720m resolution here for display purposes and classified in quantiles with each color representing 20% of the dataset.

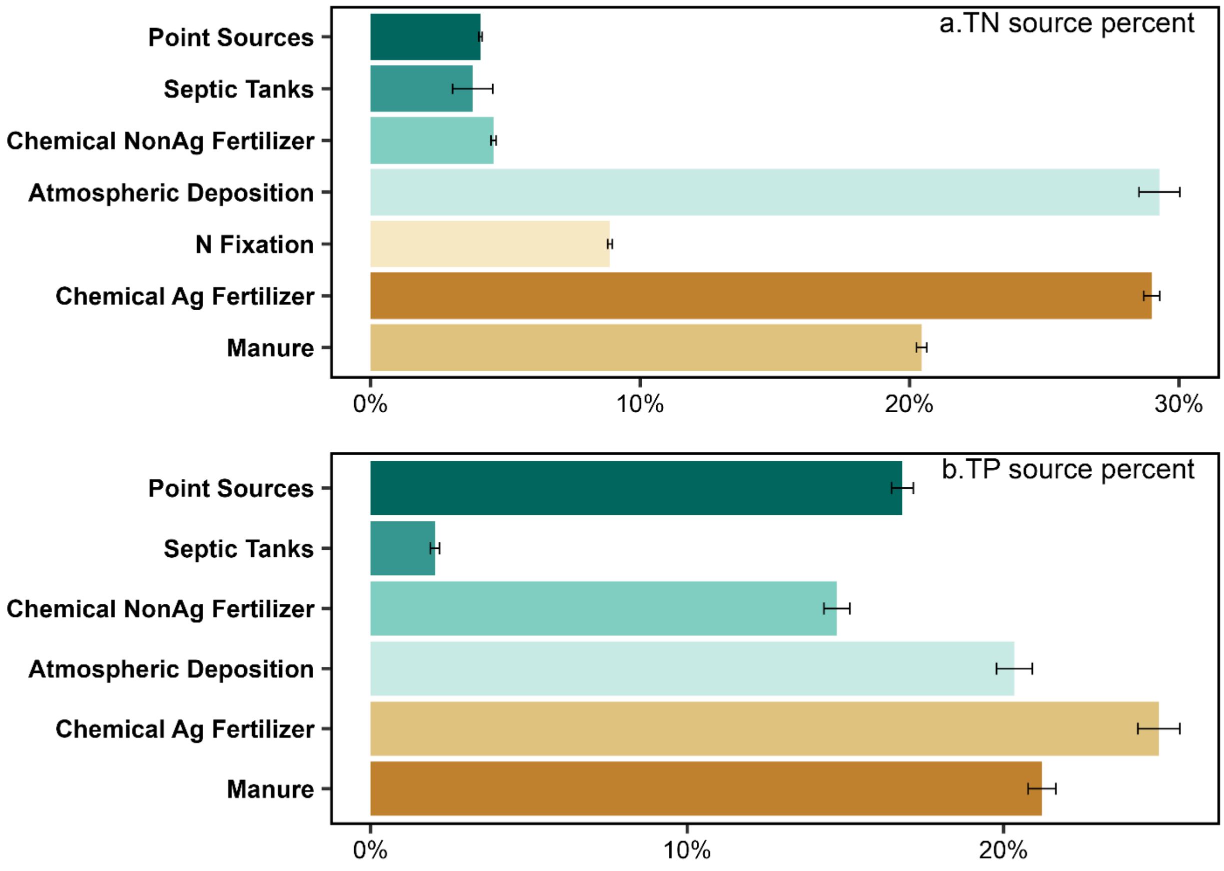

Figure S13. Estimated percentages of total nutrients (TN(a) & TP(b)) delivered to lakes by sources. Bars are the source percentage from the best-performing parameter set within the local optimization and error bars represent standard deviation from the unique best-performing global optimization runs.

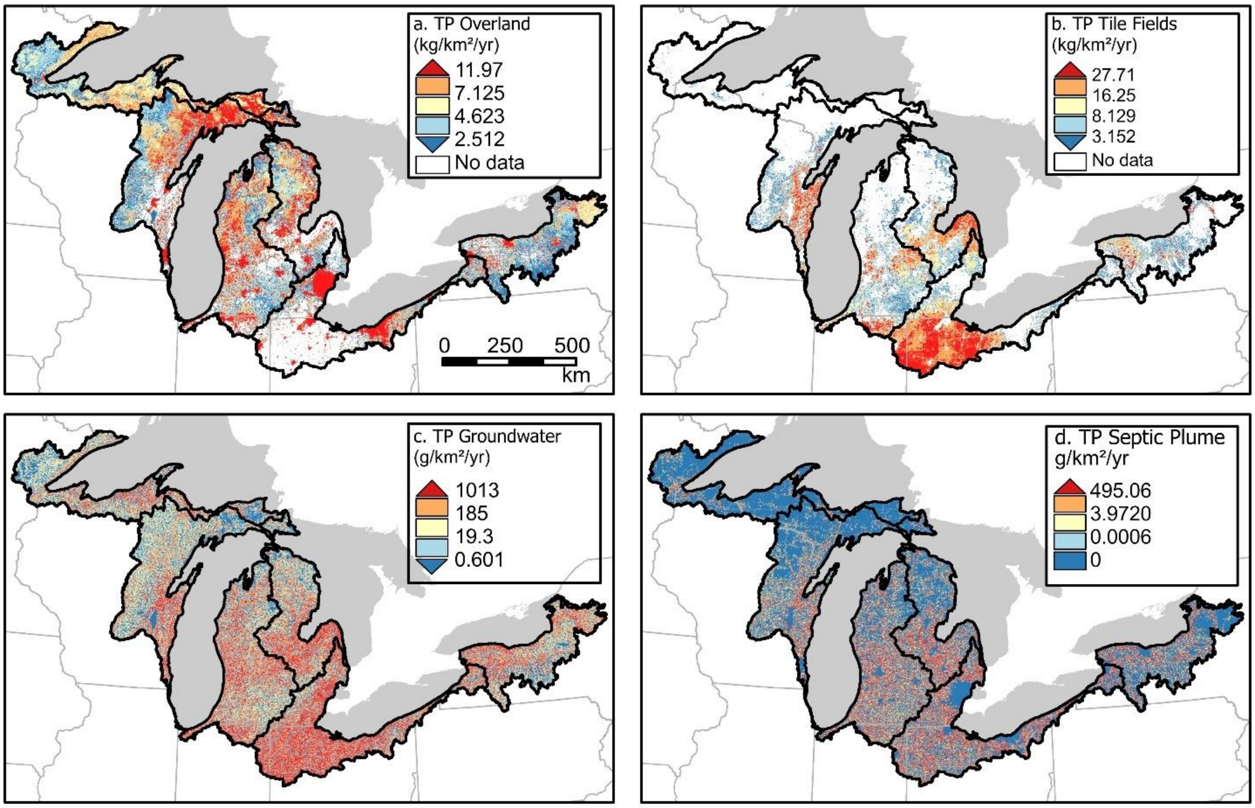

Figure S14. The estimated total yield of TP delivered to lakes by four key pathways (kg/km^2/yr). (a) - (d) for TP overland, tile fields, groundwater, and septic plume respectively. Maps are resampled from 120 m SENSEflux outputs to 720 m resolution here for display purposes and classified in quantiles, with each color representing 20% of the dataset; the white area in a&b within the basin boundary represents areas with no data as we assumed overland and tile fields are alternative pathways.

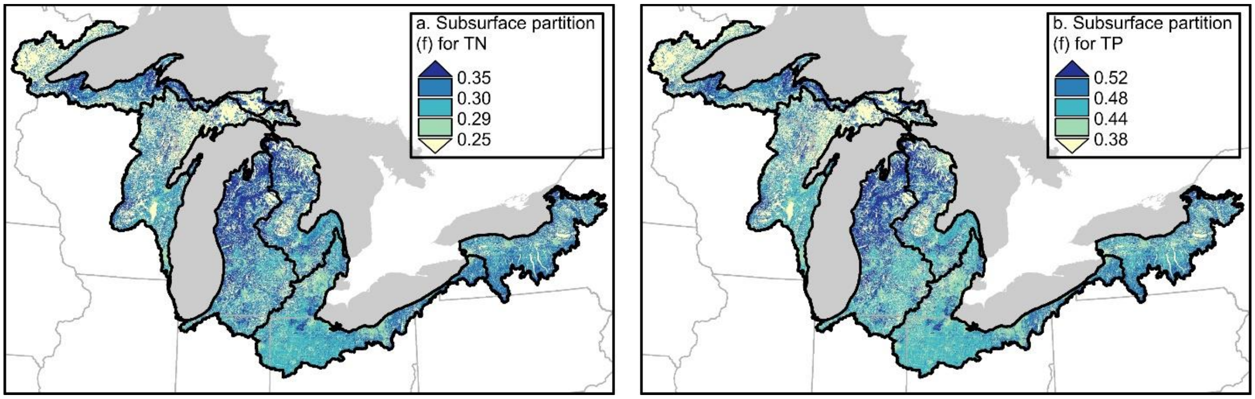

Figure S15. Spatial distribution of SENSEflux surface and subsurface partition parameter (f) for TN (a) and TP (b).

Table S1. Summary of optimized model parameters. The best-performing parameter set from local optimization is reported. The range (minimum, maximum) of the top 10% of global optimization which has lower objective function values is shown in parentheses. Note that capitalized parameter values appear directly in Equations 2 (*f*), 8 (*fstor*), 9 (*bo*), 10 (*bt*), 11 (*bg*), 12 (*bs*), 4 (*dnsp*), 5 (*bio*), 7 (*lacus*) while lower-case parameter values are input to related equations as defined in Luscz et al. (2017). One exception is a newly added parameter *fstor*, which is defined in S1.

**Function type**	**Parameter**	**General function**	**Calibrated parameter value with ranges**	
			**TN**	**TP**
Linear	f	Subsurface partition	0.49 (0.32, 0.68)	0.78 (0.76, 0.93)
	ExH	Harvest extraction	0.80 (0.72, 0.80)	0.95 (0.94, 0.95)
	SepEff	Efficiency multiplier on septic loads	0.30	0.35
	fstor	Fraction of groundwater-pathway nutrients stored in the deep unsaturated zone	0	0.55 (0.49, 0.65)
Exponential	bo	Basin attenuation (overland)	1.41E-03 (8.53E-04, 1.73E-03)	1.27E-04 (1.67E-05, 9.27E-05)
	bt	Basin attenuation (tile)	6.85E-05 (6.46E-09, 4.47E-05)	6.51E-06 (1.09E-05,2.55E-05)
	bg	Basin attenuation (groundwater)	3.76E-04 (1.16E-03, 1.74E-02)	5.90E-03 (1.81E-03, 6.28E-03)
	bs	Basin attenuation (septic)	7.12E-04 (1.46E-03, 4.65E-02)	1.91E-02 (3.33E-02, 6.88E-02)
	dnsp	N denitrification or P sorption in River	1.97E-06 (4.56E-07, 1.51E-06)	1.41E-07 (1.30E-08, 2.76E-07)
	bio	Biological Uptake and Burial in River	1.05E-04 (1.84E-06, 4.68E-05)	2.93E-04 (5.10E-05, 2.70E-04)
	lacus	Lake reduction	1.36E-05 (7.13E-06,1.22E-05)	1.94E-05 (1.81E-05, 3.70E-05)

Table S2. Total annual nitrogen and phosphorus flux and yield from the best-performing parameter set with local optimization are reported. The range of flux (kiloton per year; kt/yr) and yield (a tenth of kilogram per square kilometer per year; $kg/km^2/yr/10$) are signified by the minimum and maximum parameter value combinations within the 10% of global optimizations.

**US Great Lakes Basin**	**US drainage area (km2)**	**TN annual load (tonnes)**	**TP annual load (tonnes)**	**TN ranges**		**TP range**	
				**Flux**	**Yield**	**Flux**	**Yield**
Superior	42,199	9,214	327	7-9	17-21	0.3-0.4	0.7-0.9
Michigan	115,591	62,915	2,322	61-66	53-57	2.1-2.5	1.8-2.1
Huron	40,935	23,230	762	22-24	53-59	0.7-0.8	1.8-2.0
Erie	53,521	61,453	2,329	60-66	112-122	2.3-2.6	4.3-4.7
Ontario	34,289	17,069	564	16-19	49-53	0.4-0.6	1.4-1.8

Table S3. Range of source contributions to total basin nutrient delivery at US Great Lake Basin.

**Sources**	**TN**	**TP**
Point Sources	4% (4-5%)	17% (15-17%)
Septic Tanks	4% (0-3%)	2% (0-2%)
Chemical NonAg Fertilizer	5% (3-4%)	15% (15-17%)
Atmospheric Deposition	29% (26-29%)	20% (19-23%)
N Fixation	9% (9-10%)	—
Chemical Ag Fertilizer	29% (30-33%)	25% (24-27%)
Manure	20% (21-23%)	21% (20-23%)

Table S4. Range of pathway contributions to total nitrogen and phosphorus delivery at US Great Lake Basin.

**Pathways**	**TN**	**TP**
Point	4% (4-5%)	17% (15-17%)
Septic Plume	4% (0-3%)	2% (0-2%)
Groundwater	26% (2-17%)	5% (4-15%)
Overland	20% (15-30%)	40% (37-43%)
Tile Fields	46% (56-70%)	36% (30-37%)

Reference

(1) Luscz, E. C.; Kendall, A. D.; Hyndman, D. W. A Spatially Explicit Statistical Model to Quantify Nutrient Sources, Pathways, and Delivery at the Regional Scale. *Biogeochemistry* 2017, *133* (1), 37–57. https://doi.org/10.1007/s10533-017-0305-1.

(2) Robertson, W. D.; Stempvoort, D. R. V.; Schiff, S. L. Review of Phosphorus Attenuation in Groundwater Plumes from 24 Septic Systems. *Sci Total Environ* 2019, *692*, 640–652. https://doi.org/10.1016/j.scitotenv.2019.07.198.

(3) Hyndman, D. W.; Kendall, A. D.; Welty, N. R. H. Evaluating Temporal and Spatial Variations in Recharge and Streamflow Using the Integrated Landscape Hydrology Model (ILHM). 2007.

(4) Martin, S. L.; Hamlin, Q. F.; Kendall, A. D.; Wan, L.; Hyndman, D. W. The Land Use Legacy Effect: Looking Back to See a Path Forward to Improve Management. *Environ Res Lett* 2021, *16* (3), 035005. https://doi.org/10.1088/1748-9326/abe14c.

(5) Gleason, C. J.; Smith, L. C. Toward Global Mapping of River Discharge Using Satellite Images and At-Many-Stations Hydraulic Geometry. *Proc National Acad Sci* 2014, *111* (13), 4788–4791. https://doi.org/10.1073/pnas.1317606111.

(6) OSU. PRISM Climate Group. Oregon State University February 4, 2014. https://prism.oregonstate.edu (accessed 2020-12-16).

(7) USDA. *Soil Survey Geographic (SSURGO) Database - Drainage Class*; 2013. https://sdmdataaccess.sc.egov.usda.gov (accessed 2022-05-10).

(8) USGS. *NLCD 2011 Land Cover the Coterminous United States*. http://mrlc.gov/data.

(9) NASS. *Agricultural Census data for county-level tile drainage area, 2012*. https://www.nass.usda.gov/ (accessed 2019-02-15).

(10) Magdoff, F.; Es, H. van. Management of Nitrogen and Phosphorus. In *Building soils for better crops : ecological management for healthy soils*; Sustainable Agriculture Research and Education (SARE), 2021; pp 289–306.

(11) Prasad, R.; Chakraborty, D. *Understanding Phosphorus Forms and Their Cycling in the Soil*. https://www.aces.edu/wp-content/uploads/2019/04/ANR-2535-Phosphorus-Basics_041719L.pdf (accessed 2023-05-16).

(12) Robertson, D. M.; Saad, D. A. Nutrient Inputs to the Laurentian Great Lakes by Source and Watershed Estimated Using SPARROW Watershed Models1: Nutrient Inputs to the Laurentian Great Lakes by Source and Watershed Estimated Using SPARROW Watershed Models. *Jawra J Am Water Resour Assoc* 2011, *47* (5), 1011–1033. https://doi.org/10.1111/j.1752-1688.2011.00574.x.

(13) Hamlin, Q. F.; Kendall, A. D.; Martin, S. L.; Whitenack, H. D.; Roush, J. A.; Hannah, B. A.; Hyndman, D. W. Quantifying Landscape Nutrient Inputs With Spatially Explicit Nutrient Source Estimate Maps. *J Geophys Res Biogeosciences* 2020, *125* (2). https://doi.org/10.1029/2019jg005134.
